# STAT3 Inhibition Attenuates MYC Expression by Modulating Co-Activator Recruitment and Suppresses Medulloblastoma Tumor Growth by Augmenting Cisplatin Efficacy In Vivo

**DOI:** 10.3390/cancers15082239

**Published:** 2023-04-11

**Authors:** Kyle A. Rohrer, Heyu Song, Anum Akbar, Yingling Chen, Suravi Pramanik, Phillip J. Wilder, Erin M. McIntyre, Nagendra K. Chaturvedi, Kishor K. Bhakat, Angie Rizzino, Don W. Coulter, Sutapa Ray

**Affiliations:** 1 Department of Pediatrics, Hematology and Oncology Division, Nebraska Medical Center, Omaha, NE 68198, USA; 2 Department of Genetics, Cell Biology and Anatomy, University of Nebraska Medical Center, Omaha, NE 68198, USA; 3 Department of Medicine, University of Arizona, Tucson, AZ 85721, USA; 4 Child Health Research Institute, University of Nebraska Medical Center, Omaha, NE 68198, USA; 5 Eppley Institute for Research in Cancer and Allied Diseases, Omaha, NE 68198, USA; 6 Fred & Pamela Buffett Cancer Center, Omaha, NE 68198, USA

**Keywords:** medulloblastoma, STAT3, MYC, p300, H3K27Ac, chemoresistance

## Abstract

**Simple Summary:**

Medulloblastoma (MB) is a malignant brain tumor of childhood that occurs in the cerebellum and accounts for 20–25% of all pediatric central nervous system tumors. Despite improvement in overall survival rate, it still lacks an effective targeted treatment strategy. Here, we delineated mechanistically the functional role of activated signal transducers and activators of transcription-3 (STAT3) in the expression of oncogenic targets MYC, and in promoting MB tumorigenesis and chemoresistance. We validated the in vitro and in vivo efficacy of inhibiting STAT3 by genetic and pharmacologic inhibition in combination with chemotherapy in subcutaneous and orthotopic mouse models of MB. STAT3 inhibitors, along with chemotherapy, may serve as a novel therapeutic strategy, improving outcomes and the quality of life in pediatric MB patients.

**Abstract:**

MB is a common childhood malignancy of the central nervous system, with significant morbidity and mortality. Among the four molecular subgroups, MYC-amplified Group 3 MB is the most aggressive type and has the worst prognosis due to therapy resistance. The present study aimed to investigate the role of activated STAT3 in promoting MB pathogenesis and chemoresistance via inducing the cancer hallmark MYC oncogene. Targeting STAT3 function either by inducible genetic knockdown (KD) or with a clinically relevant small molecule inhibitor reduced tumorigenic attributes in MB cells, including survival, proliferation, anti-apoptosis, migration, stemness and expression of MYC and its targets. STAT3 inhibition attenuates MYC expression by affecting recruitment of histone acetyltransferase p300, thereby reducing enrichment of H3K27 acetylation in the MYC promoter. Concomitantly, it also decreases the occupancy of the bromodomain containing protein-4 (BRD4) and phosphoSer2-RNA Pol II (pSer2-RNAPol II) on MYC, resulting in reduced transcription. Importantly, inhibition of STAT3 signaling significantly attenuated MB tumor growth in subcutaneous and intracranial orthotopic xenografts, increased the sensitivity of MB tumors to cisplatin, and improved the survival of mice bearing high-risk MYC-amplified tumors. Together, the results of our study demonstrate that targeting STAT3 may be a promising adjuvant therapy and chemo-sensitizer to augment treatment efficacy, reduce therapy-related toxicity and improve quality of life in high-risk pediatric patients.

## 1. Introduction

Medulloblastoma (MB), the most common brain tumor of the cerebellum, is a leading cause of cancer-related deaths in children. The current standard of care for MB patients involves a combination of maximal surgical resection followed by craniospinal radiation and high-dose chemotherapy [1,2]. However, these therapies are associated with long-term neurocognitive sequelae and patients with recurrent disease have an increased risk of mortality [3]. MB tumors are highly heterogeneous and are classified into four major molecularly distinct subgroups: WNT (Wingless), SHH (Sonic Hedgehog), Group 3 and Group 4 [4,5,6]. Frequent amplification of the MYC oncogene is found in all MB subgroups; of particular note, Group 3 MB with c-MYC amplification was identified as having the worst clinical outcomes with a 10-year survival rate <20% [7]. Clearly, molecularly targeted therapy that is more effective and less toxic is needed to ameliorate treatment-related side-effects in high-risk MB patients [8]. 

Activation of the STAT3 transcription factor by phosphorylation at Y705 is transient in normal cells [9,10,11]. However, STAT3 is aberrantly activated by increased phosphorylation in nearly 70% of human cancers, including brain tumors and MB. Activated STAT3 drives multiple pro-oncogenic functions via expression of its target genes that promote uncontrolled cellular proliferation, survival, angiogenesis and chemoresistance, and, therefore, it is widely considered as an oncogene [7,12]. In cancer, sustained STAT3 activation occurs through several mechanisms, e.g., excessive cytokine and growth factor stimulation, hyper-activation or mutation of receptor/non-receptor associated kinases (JAK and Src family kinases), or loss of negative regulators (SOCS and PIAS proteins) [7,13]. STAT3 negative regulators tightly control its transcriptional activity under normal conditions. However, our earlier study showed that PIAS3 (protein inhibitor of activated STAT3) is repressed in MB by a non-coding onco-miR-21, leading to persistent STAT3 activation [7,14,15]. Such abnormal activation of STAT3 often leads to malignant cellular transformation and, therefore, it is considered as a potential target molecule for cancer therapy [16]. 

Overexpression/or amplification of the MYC gene has been detected in numerous solid tumors, including MB [17,18,19]. MYC is also an important downstream effector of the STAT3 pathway that is involved in cell growth, transformation and chemoresistance [20,21,22,23]. However, it remains unexplored how activated STAT3 signaling regulates and contributes to MYC expression and induces MB pathogenesis and resistance to chemotherapy. Here, we performed a detailed mechanistic study using the dual approaches of inducible genetic KD and pharmacologic inhibition of STAT3 on MYC gene regulation, MB tumor growth and chemosensitivity. Using ChIP-qPCR, we demonstrate that STAT3 inhibition affects the chromatin landscape of the MYC promoter and reduces pSer2-RNAPol II occupancy, resulting in repression of transcription [24]. Importantly, we show that STAT3 KD or inhibition with WP1066 attenuates MB tumor growth in a subcutaneous xenograft and in an orthotopic MB model and sensitizes MB tumor to cisplatin chemotherapy. Overall, our study demonstrates that STAT3 inhibition and concomitant downregulation of MYC have a significant regulatory effect on MB tumorigenesis and chemoresistance. Thus, STAT3 inhibitors can be used as a potential adjuvant therapy to treat high-risk MB patients, reducing chemotherapy-related side-effects, and eventually improving the quality of life of pediatric patients. 

## 2. Materials and Methods

### 2.1. Cell Lines and Reagents

The MB cell line HD-MB03 (MYC-amplified) was purchased from Deutsche Sammlung von Mikroorganismen und Zellkulturen (Germany) and the MB cell line ONS-76 (non-MYC-amplified) was purchased from Sekisui-XenoTech (Kansas City, KS, USA). All cell lines were authenticated by their respective resources using short tandem repeat DNA profiling and were tested for mycoplasma contamination using the MycoSensor PCR Assay Kit (Agilent Technologies; Santa Clara, CA, USA). All cell lines were cultured and maintained using RPMI-1640 media supplemented with 10% heat-inactivated FBS and 1% penicillin-streptomycin in a humidified incubator at 5% CO_2_ and 95% air atmosphere at 37 °C. 

For inducible knockdown of STAT3, control shRNA (Catalog ID: RHS4743) and STAT3 shRNA clone ID: V3THS_376016 (ATAGTTGAAATCAAAGTCA) and clone ID _376017 (AAGTTTCTAAACAGCTCCA) were purchased from Horizon (previously Dharmacon, Lafayette, CO, USA). Parental cell lines HD-MB03 and ONS-76 were engineered for Dox-inducible expression of STAT3 shRNA (with ID_376016 and 376017) or a non-targeting TRIPZ shRNA (designed with minimal homology to known mammalian genes). The TRIPZ vector results in puromycin resistance and constitutive expression of a reverse tet transactivator as well as Dox-inducible expression of the shRNA and red fluorescent protein (RFP). Engineered cells HD-shSTAT3 and ONS-shSTAT3 were either selected with 5 μg/mL puromycin (P8833, Sigma-Aldrich, St. Louis, MO, USA) for 3–5 days and/or selected for RFP-expressed cells by flow cytometry. Although both STAT3 shRNAs (ID_376016 and 376017) effectively KD STAT3 in both cell lines, we used HD-shSTAT3 (ID_376017) and ONS-shSTAT3 (ID_376016) cells in our experimental studies. IL6 and soluble IL6 receptor alpha were purchased from PeproTech Inc. (Rocky Hill, NJ, USA) [25,26,27]. Doxycycline (Dox) was purchased from Sigma Aldrich (St. Louis, MO, USA) and resuspended in water and stored at −20 °C. WP1066 was purchased from Selleckchem (Houston, TX, USA) and resuspended in DMSO and stored at −20 °C. Cisplatin, purchased from Sigma Aldrich, (St. Louis, MO, USA) was resuspended in 0.9% NaCl and stored at 4 °C [14].

### 2.2. MTT Assay

A total of 6–8 × 10^3^ cells were plated and cultured in 100 μL media containing indicated concentrations of drugs or vehicle in 96-well plates, as per the experimental design. The viability of these cells was determined at 48 h using MTT assay (ATCC; 30-1010K) following the manufacturer’s protocol. The absorbance was detected at 570 nm wavelength using a plate reader (Bioteck, Winooski, VT, USA).

### 2.3. Apoptotic Assay and Cell Cycle Analysis

The effects of STAT3 KD of MB cells on apoptosis induction and cell-cycle analysis were determined using Annexin V-FITC double staining (cat #130-092-052, MACS, Auburn, CA, USA) and by propidium iodide (PI) staining (Abcam, cat # ab139418, Cambridge, MA, USA), respectively, as previously described [14,28]. 

### 2.4. Western Blot Analysis

HD-shSTAT3 and ONS-shSTAT3 cells were treated with Dox for the indicated time and whole cell extracts (WCE) were made using RIPA buffer. Western blot analysis was performed using a standardized protocol as described previously [29,30]. Appropriate primary and secondary antibodies were used to probe the membrane following the manufacturer’s instructions. Signals were detected by enhanced chemiluminescence and using a MyECL imager (Thermo-Scientific, Waltham, MA, USA). The primary antibodies (Abs) used in WB were purchased from Cell Signaling Technology (CST, Danvers, MA, USA) or Santa Cruz Biotechnology (Dallas, TX, USA). These included: STAT3 (CST # 4904S), pY705-STAT3 (CST #9145), pS727-STAT3 (CST # 9134S), MYC (CST #18583S), BCL-XL (CST #2764S), BRD4 (CST #13440S), CCND1 (CST #55506S), β-Actin (sc-47778), GAPDH (CST #5174S), cyclophilin B (CST # 43603S), histone H3 (CST # 9715S), H3K27Ac (CST # 4353S), CD133 (CST # 64326S), Oct3/4 (sc-5279), nestin (CST # 33475S), and Sox2 (CST # 3579S).

### 2.5. RNA-seq and Quantitative PCR (qPCR)

HD-shSTAT3 and ONS-shSTAT3 cells were treated with various concentrations of Dox for 24–48 h. Total RNA was prepared using the trizol reagent (Ambion, Grand Island, NY, USA) and RNA quality and integrity were measured prior to qPCR. A total of 2 μg RNA was used for reverse transcription using the SuperScript VILO cDNA kit (Invitrogen, Carlsbad, CA, USA). cDNA products were amplified in 10 μL reactions using SYBR Green Super Mix (Applied Biosystems, Foster City, CA, USA) and validated gene-specific primers, purchased from Integrated DNA Technologies (IDT) (Coralville, IA, USA). All reactions were processed in a Quant Studio 3 real-time PCR system and the results were analyzed by data analysis software (Applied Biosystems). For RNA sequencing, RNAs from Dox-treated MB cells (in triplicates) were purified using the Qiagen RNeasy Kit. After confirming the sequence grade quality of RNA using an Agilent 2100 Bioanalyzer, an RNA library was prepared using a True-Seq RNA Sample Prep V2 Kit and subjected to RNA sequencing using the Illumina NextSeq550 system in the UNMC Genomics Core Facility. The Tuxedo pipeline [31] was used to find differentially expressed genes (DEGs). The TopHat software package was used to align the reads to the human genome. Cufflinks was used to determine FPKM (fragments per kilobases of transcripts per million mapped reads) values for each transcript in each sample.

### 2.6. Cell Migration Assays

MB cells were cultured in a monolayer and then wounded using 200 μL pipet tips. Cells were then treated either with Dox or left untreated. The migration distance was photographed and measured at time zero and after 36 h [14].

### 2.7. Colony Assay

MB cells were treated with Dox for 48 h and then with cisplatin prior to reseeding in six well plates (400–800 cells/well). Cells were grown in normal media for about 12–14 days. Colonies were fixed in acetic acid/methanol (1:7) for 15 min and then stained with 0.5% crystal violet for 10 min. Cells were washed with water and air-dried overnight before taking pictures. Colonies were counted and are shown in the bar graph.

### 2.8. MB-Tumorsphere Assay

Cells (~10,000 cells) were plated in ultra-low attachment 6-well plates in serum-free media containing EGF 20 ng/mL, bFGF 40 ng/mL, heparin 2 mg/mL, B-Me 0.1 mM, B27 1%, N_2_ and Pen-Strep 1% for 10–14 days. Alternatively, spheres were treated with either Dox or left untreated and sphere numbers and sizes were counted and images were taken in an EVOS cell imaging system. WCEs from the spheres were also prepared from the above treatment for determining the expression of the neural stem cell markers. 

### 2.9. Chromatin Immunoprecipitation (ChIP)-qPCR Assay

HD-shSTAT3 cells were treated either with Dox for 48 h or left untreated. ChIP assay was performed as previously described [14,32] using STAT3, BRD4, pSer2-RNApol II, H3K27Ac and p300 Abs. Genomic qPCR was performed in a Quant Studio 3 system, using SYBR green. ChIP primers for human MYC promoter (#14905) and CCND1 promoter (#12531) were purchased from Cell Signaling Technology (CST, Danvers, MA, USA). The sequences of MYC primers used were: forward primer (5′->3′): TGAGTATAAAAGCCGGTTTTCG; reverse primer (5′->3′): CTGCCTCTCGCTGGAATTACTA. Data are presented by normalizing with either percent input DNA or IgG control. 

### 2.10. In Vivo Animal Xenograft

(A) Subcutaneous:

All animal experiments were performed according to a UNMC Institutional Animal Care and Use Committee-approved protocol, 19-081-03-FC. For the subcutaneous xenograft study, 5–6-weeks-old nude mice were purchased. Suspensions of 0.5 × 10^6^ MB cells (HDMB-shSTAT3 and ONS-shSTAT3 cells) were mixed with an equal volume of Matrigel and were injected into both flanks of nude mice. Once a palpable tumor had formed, mice were given either Dox (2 mg/mL in drinking water) or cisplatin (2 mg/kg; twice a week) intra-peritoneally (i.p.) alone or in combination for four weeks. Tumor growth was evaluated by electronic caliper twice a week, and the tumor volumes were calculated as length × width^2^ × 0.5. To observe the effects of the STAT3 small molecule inhibitor WP1066 on MB tumor growth and chemosensitivity, HD-MB03 cells were subcutaneously injected into nude mice as above, and WP1066 treatment (30 mg/kg, i.p., 3 times a week for 4 weeks) was initiated when the tumors reached ~100–150 mm^3^. Cisplatin (2 mg/kg) treatment was also given twice a week for 4 weeks and tumor size was evaluated twice a week as mentioned above. To determine the effect of STAT3 inhibition on the survival of tumor-bearing mice, 4 weeks after the treatment was terminated, tumor growth was monitored further for another 4 weeks. Mice were euthanized by cervical dislocation when tumor size reached greater than 1 cm^3^. Tumor tissues were processed for histological and IHC analyses to determine the tumor burden. Survival of the vehicle or drug treated mice were analyzed by the Kaplan–Meier method [33].

(B) Intracranial:

Immunocompromised NOD-scid IL2Rgammanull (or NSG) mice, 6–8 weeks old, purchased from the Jackson Laboratory were used for orthotopic xenograft studies. HD-MB03 cells expressing RFP/luciferase (2.5 × 10^5^) were suspended in 3 μL neural stem cell media and injected into the cerebella (2 mm posterior to the lambda suture, 2 mm lateral to the midline, and 2.5 mm deep) of mice brains using a Hamilton neuro-syringe (33G) attached to the stereotaxic apparatus (David Kopf Instruments, Tujunga, CA, USA). Intracranial xenografts in mice were detected by IVIS imaging by injecting d-luciferin (70 mg/kg) at day 5 after implantation. Mice with a detectable signal were divided into four groups (*n* = 5): vehicle, cisplatin, WP1066, and the combination of WP1066 plus cisplatin, and treatment doses were given as above intra-peritoneally every other day for 16 days (9 total treatments). Mice were imaged on the IVIS2000 system (PerkinElmer, Waltham, MA, USA) at day 5 and at day 21. Mice from each group were sacrificed on day 21 and their brains were harvested. H&E staining and IHC analyses of Ki67 were performed to confirm the histology and proliferation of tumor cells.

### 2.11. Immunofluorescence (IF) and Immunohistochemistry (IHC)

For IF, cells grown on coverslips were fixed with 4% formaldehyde (Sigma Aldrich, St. Louis, MO, USA) and stained as described previously [34,35]. The primary antibody used for IF was anti-H3K27Ac (1:50; CST #4353S). Images were acquired by fluorescence microscope with a 63 X oil immersion lens (LSM510; Zeiss, White Plains, NY, USA). MB xenograft sections of vehicle- and drug-treated groups were subjected to IHC staining with pTyr705-STAT3, caspase3, Ki67 and MYC Ab following the manufacturer’s instructions, at the University of Nebraska Medical Center (UNMC) Tissue Science Facility. 

### 2.12. Statistical Analysis

All analyses were performed using GraphPAD Prism7. The results are representative of three independent experiments and are expressed as the means ± SD, as indicated. Statistical analysis of the data was performed using the two-tailed unpaired Student’s *t*-test or one-way ANOVA. Survival of xenograft mice was estimated with Kaplan–Meier survival analysis and significance was determined with a log-rank test (Mantel–Cox). A *p* value less than 0.05 was considered statistically significant. To determine synergy, we employed the Chou and Talalay method for combination index (CI) analysis; CI < 1 indicates synergism, CI = 1 additive and CI > 1 antagonism [36].

## 3. Results

### 3.1. Doxycycline-Inducible STAT3 shRNA Decreases Endogenous STAT3 and STAT3 Targets

**Figure 1:**
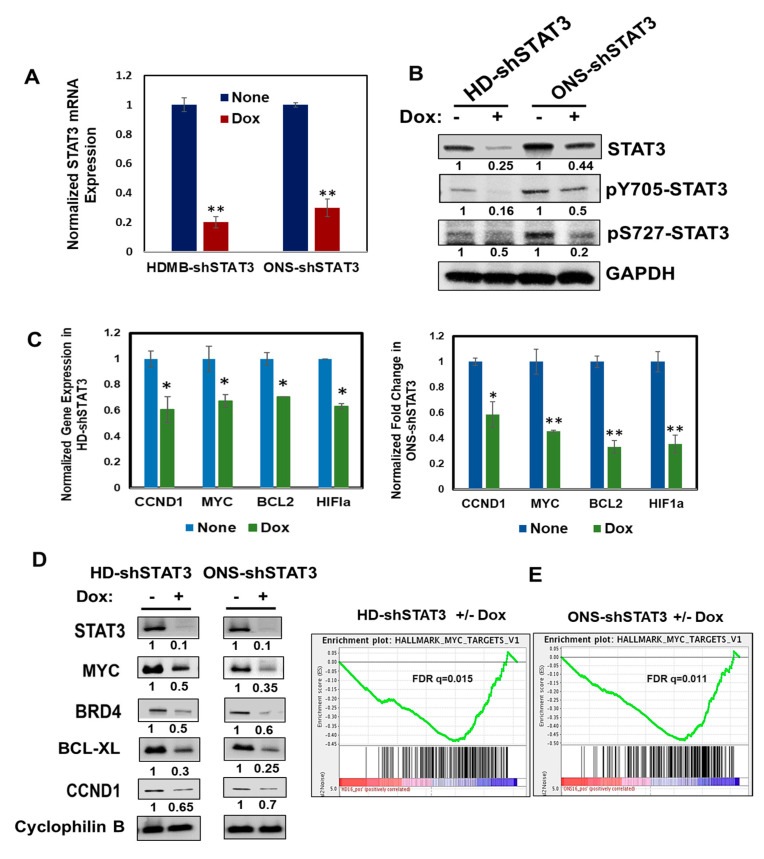
Effect of STAT3 genetic KD on STAT3 targets in vitro. (**A**) STAT3 mRNA expression in HD-shSTAT3 and ONS-shSTAT3 MB cells expressing Dox-inducible STAT3 shRNA was validated by qPCR. Cells were either treated with Dox (0.5 μg/mL) for 48 h or left untreated before harvesting the RNA. ** Represents *p* < 0.01 for Dox-treated cells versus control cells. (**B**) HD-shSTAT3 and ONS-shSTAT3 MB cells were treated as in (**A**) prior to making the whole cell extract (WCE). Expression levels of STAT3, pY705-STAT3 and pS727-STAT3 were analyzed by Western immunoblot analysis and GAPDH was used as a loading control. (**C**) Cells were treated as in (**A**) and STAT3 target gene expression was analyzed by qPCR. * Represents *p* < 0.05, ** *p* < 0.01. (**D**) WCEs from Dox-treated HD-shSTAT3 and ONS-shSTAT3 MB cells were analyzed by Western blot for the expression levels of STAT3 target genes. Cyclophilin B acts as a loading control. (**E**) Enrichment of MYC target genes is shown in HD-shSTAT3 and ONS-shSTAT3 cells after STAT3KD by GSEA plot. FDR represents false discovery rate; FDR q < 0.25 is considered significant. The uncropped bolts are shown in Supplementary Materials file S1.

To access the functional role of STAT3 in MB tumorigenesis, we used the parental MB cells HD-MB03 (MYC amplified and overexpressed) and ONS-76 (single copy MYC) and generated doxycycline (Dox)-inducible HD-shSTAT3 (shRNA ID_376016 and 376017) and ONS-shSTAT3 cells (shRNA ID_376016), respectively, that co-express STAT3 shRNA and a red fluorescence protein (RFP) in the presence of Dox, as shown in Figure S1. Further, Dox treatment in each STAT3-shRNA-inducible cell line significantly decreased STAT3 mRNA and protein expression compared to untreated controls in qPCR and Western blot, respectively (Figure S2A,B). Although both STAT3 shRNAs (ID_376016 and 376017) effectively KD STAT3 in MB cells, we used HD-shSTAT3 (ID_376017) and ONS-shSTAT3 (ID_376016) cells in our experimental studies. In Figure 1A,B, Dox treatment in HD-shSTAT3 and ONS-shSTAT3 cells show downregulation of STAT3 mRNA and total and phosphorylated STAT3 levels in qPCR and Western blot, respectively. Although HD-shSTAT3 and ONS-shSTAT3 cells downregulated STAT3 expression in the presence of increasing doses of Dox (Figure S3), the parental cell lines (HDMB-03 and ONS-76), or the non-targeting shRNA cells (HD-shNTC), did not show any change in STAT3 protein expression or STAT3 target gene expression (Figure S4). Next, we determined, by qPCR (Figure 1C) and Western blots (Figure 1D), how STAT3 KD affected expression of STAT3 target genes, including MYC. We also performed RNA sequencing using HD-shSTAT3 and ONS-shSTAT3 cells and compared the gene expression profiles before and after Dox treatment to knockdown STAT3. The differential gene expression heat map from Euclidean cluster analysis showed a significant (FDR ≤ 0.05) and log2-fold change ≥ 1 or ≤ −1 from control to treatment (−/+ Dox) in a minimum of one comparison (Figure S5). Furthermore, gene-set enrichment analyses (GSEA) demonstrated marked enrichment for biological processes regulated by STAT3 (Figure S6); in particular, it significantly downregulated MYC targets, indicating that STAT3 KD has profound effects on its target genes, including MYC, a hallmark of cellular proliferation (Figure 1E) [37].

### 3.2. STAT3 KD Affects Cell Viability, Apoptosis, Cell Growth and Migration

**Figure 2:**
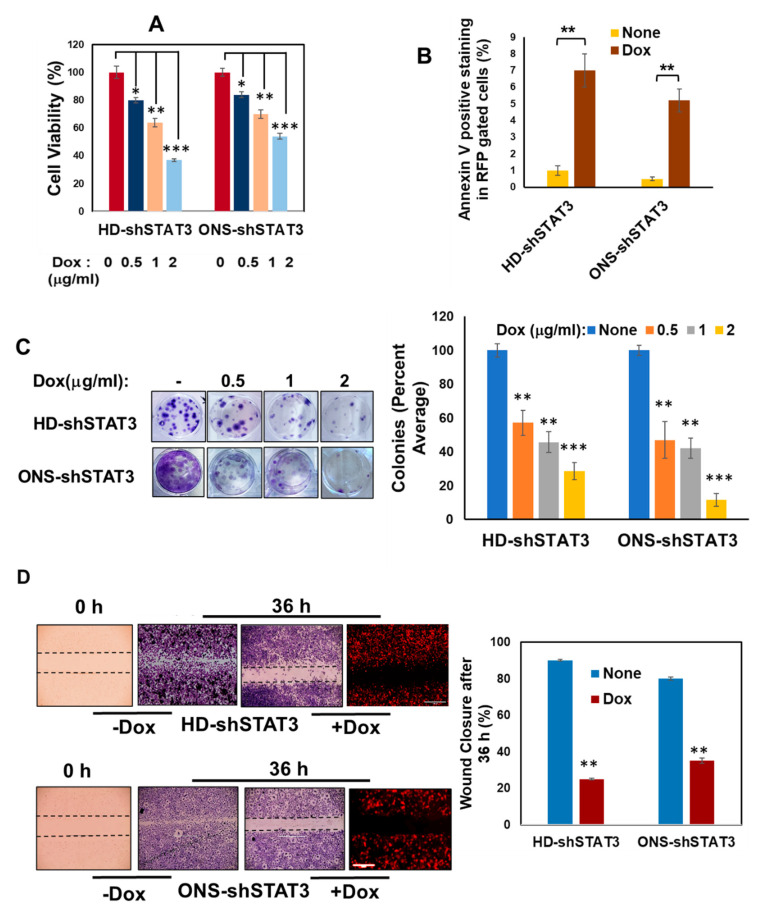
Effect of STAT3 KD on cell viability, apoptosis, cell growth and migration. (**A**) HD-shSTAT3 and ONS-shSTAT3 MB cells were treated either with Dox at a concentration of 0.5, 1, or 2 μg/mL or left untreated. The growth of these cells was determined at 48 h using MTT assays. The values represent the means ± SD from five wells of a 96-well plate; * *p* < 0.05, ** *p* < 0.01 and *** *p* < 0.001. (**B**) MB cell lines were treated either with Dox at a concentration of 0.5 μg/mL for 48 h or left untreated. RFP expressing cells were gated and the percentage of cells undergoing apoptosis was determined using an annexin-V-FITC apoptosis detection kit. Representative figure shows a bar graph of quantification of the apoptotic cells from one of three independent experiments; ** *p* < 0.01. (**C**) Equal numbers of cells (~400) were seeded in 6-well plates and were treated with either 0.5, 1, or 2 μg/mL of Dox or left untreated for 10–14 days. Colonies formed were fixed with acetic acid/methanol 1:7 (*v*/*v*) and stained with 0.5% crystal violet solution. Number of colonies were counted from triplicate wells and are shown in bar diagram (right); ** *p* < 0.01 and *** *p* < 0.001. (**D**) Wound-healing assays were performed on cells seeded into 6-well assay plates until a monolayer formed. A cell-free gap/scratch was created in which the cell migration was analyzed in the presence of 0.5 μg/mL Dox. Representative images of cell migration are shown after 36 h; ** *p* < 0.01.

To assess the effects of STAT3 genetic KD on MB cell growth, we investigated the effect of Dox by MTT assay. After treatment with increasing concentration of Dox for 48 h, HD-shSTAT3 and ONS-shSTAT3 cell growth was significantly reduced in a dose-dependent manner (Figure 2A), whereas the parental cell lines (HDMB-03 and ONS-76) and HD-shNTC did not show any change in cell viability (Figure S7). Annexin V staining in HD-shSTAT3 and ONS-shSTAT3 MB cells followed by flow cytometry showed 7% and 5.2% apoptotic cell death in Dox-treated cells, respectively, compared to untreated controls (Figure 2B). Further, we found that STAT3 KD induced cell-cycle arrest at the G2 phase, both in HD-shSTAT3 (~2% increase) and ONS-shSTAT3 (~1.5% increase) cells compared to Dox-untreated cells (Figure S8). Next, we performed a clonogenic assay to measure the proliferative capability of STAT3 KD cells to form colonies from a single cell. As shown in Figure 2C, with increasing concentration of Dox, the numbers of colonies were significantly reduced in HD-shSTAT3 and ONS-shSTAT3 MB cells expressing STAT3 shRNA. These data indicate that STAT3 inhibition slows growth in vitro and inhibits the ability of MB cells to form colonies. Cell migration is an important step in the metastatic capability of cancer cells. We evaluated the effect of STAT3 KD on cell migration using a scratch wound assay [38]. We observed that untreated HD-shSTAT3 and ONS-shSTAT3 cells migrated to fill 90% and 85% of the gap area within 36 h, whereas Dox-treated STAT3 KD cells took a significantly longer time to fill only 22% and 37% of the scratch area (Figure 2D). These data suggest that reduced STAT3 expression affects the migratory properties of MB cells and likely their ability to metastasize.

### 3.3. Effect of STAT3 KD on MB Sphere Formation

**Figure 3:**
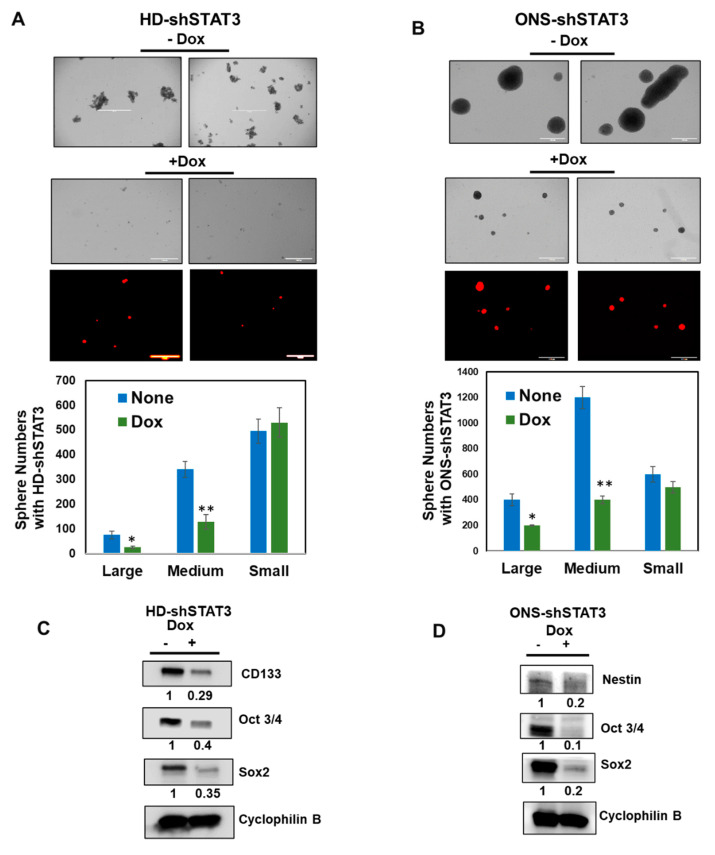
Effect of STAT3 KD on MB tumorsphere formation. (**A**,**B**) show representative morphologies of MB tumorspheres from HD-shSTAT3 and ONS-shSTAT3 MB cells grown in serum-free media in the presence and absence of 0.5 μg/mL Dox. MB spheres with RFP expression in the presence of Dox are also shown (bottom). Small, medium and large spheres from the cells were enumerated and are shown below in the bar graph; * *p* < 0.05, ** *p* < 0.01. (**C**,**D**) show the expression of neural stem cell markers in the absence and presence of Dox in HD-shSTAT3 and ONS-shSTAT3 cells by Western immunoblot. Cyclophilin B acts as a loading control. The uncropped bolts are shown in Supplementary Materials file S1.

A small subset of cells within a tumor, known as cancer stem cells, possess stem-cell-like properties. These cells are thought to be responsible for MB tumor progression and relapse [39]. As STAT3 is known to play an important role in promoting self-renewal of cancer stem cell enrichment and to give rise to tumorspheres, we performed a tumorsphere formation assay in vitro with HD-shSTAT3 and ONS-shSTAT3 cells grown in serum-free medium in ultra-low adherent tissue culture plates [40,41]. We found that both HD-shSTAT3 and ONS-shSTAT3 cells formed MB-tumorspheres of varied numbers and sizes, whereas, in the presence of Dox, the number and size of MB tumorspheres were reduced significantly (Figure 3A,B). We further tested the effect of STAT3 KD on the expression of neural stem cell markers in MB-tumorsphere extracts by Western blot analyses. Figure 3C,D shows the expression of CD133, Sox2 and Oct3/4 in HD-shSTAT3 cells and the expression of nestin, OCT3/4 and Sox2 in ONS-shSTAT3 cells, respectively, prior to Dox treatment. In contrast, in the presence of Dox, expression of these markers was reduced, indicating STAT3 is essential for MB sphere formation and the expression of genes associated with cancer stem cells.

### 3.4. STAT3 KD Attenuates Target Gene Expression by Affecting Coactivators and RNA Pol II Recruitment

**Figure 4:**
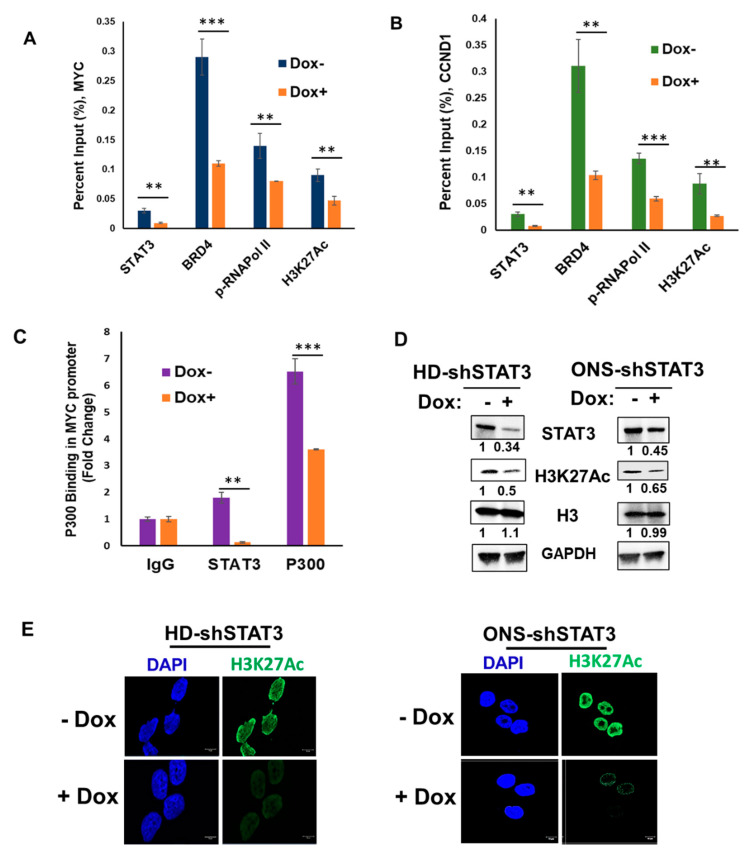
STAT3 KD attenuates histone modification and RNA Pol II recruitment on target gene promoters. MB cells were treated with 0.5 μg/mL Dox prior to crosslinking with formaldehyde. STAT3, BRD4, pSer2-RNAPol II, and H3K27Ac binding to the MYC (**A**) and CCND1 (**B**) promoters were demonstrated by ChIP-qPCR. IgG Ab was used as a negative control and data are presented as percent (%) input; ** *p* < 0.01, *** *p* < 0.001. (**C**) Cells were treated the same as before; binding of STAT3 and p300 on the MYC promoter are presented as fold change compared to IgG; ** *p* < 0.01, *** *p* < 0.001. (**D**) HD-shSTAT3 and ONS-shSTAT3 MB cells were treated either with or without Dox and WCEs were analyzed for H3K27Ac expression. Total H3 and GAPDH were used as control. (**E**) Cells were grown in coverslips and were treated as in (**D**) and an immunofluorescence (IF) experiment was performed to detect the levels of H3K27Ac. Nuclear staining is shown by DAPI. The uncropped bolts are shown in Supplementary Materials file S1.

To understand the mechanism by which STAT3 inhibition attenuates MB cell proliferation, we examined the binding of STAT3 to its downstream target gene promoters by ChIP assay. We found that, in the presence of Dox, the abundance of STAT3 in the MYC and CCND1 (Figure 4A,B) promoters was significantly reduced in MB cells compared to untreated control. We previously showed that activated STAT3 recruits BET family member BRD4, an important transcriptional co-regulator and an epigenetic reader of many genes, including MYC [27]. Therefore, we examined the occupancy of BRD4 and pSer2-RNAPol II, as BRD4 is known to be physically associated with elongating Pol II complexes [42]. As expected, we observed decreased BRD4 and pSer2-RNAPol II loading in the MYC and CCND1 promoters with STAT3 KD in HD-shSTAT3 MB cells (Figure 4A,B). Moreover, as BRD4 binds acetylated histone tails at lysine 27, we next used antibodies against H3K27Ac in the MYC promoter. Although acetylation of H3K27 histone modification is typically found at the enhancers, publicly available data on the Cistrome data browser indicates that the H3K27 is acetylated at the MYC promoter in HCT-116 (colorectal carcinoma) and in A549 (lung adenocarcinoma) cells. Indeed, our findings indicate that acetylation of H3K27 at the MYC and CCND1 promoters was reduced upon STAT3 KD (Figure 4A,B). To understand if low enrichment of the H3K27Ac mark on the promoters was due to reduced binding of p300 (a broad-spectrum histone acetyl transferase which acetylates histone H3 at K27), [43] we examined the occupancy of p300 on the MYC gene and found that its binding to the promoter sequence was significantly decreased (Figure 4C). This indicated that STAT3-mediated recruitment of the transcriptional machinery and subsequent enrichment of H2K27Ac are necessary for proper execution of transcription and that KD of STAT3 impairs cooperative DNA binding, thereby attenuating gene expression in MB. We also determined if STAT3 KD affects change in H3K27Ac level in the HD-shSTAT3 and ONS-shSTAT3 cells. Western blot and immunofluorescence (IF) data showed that Dox treatment decreased H3K27Ac levels in both types of cells, further indicating a specific role of STAT3 in histone modification and chromatin accessibility of the transcription machinery (Figure 4D,E). 

### 3.5. STAT3 KD Increases Sensitivity of MB Cells to Chemotherapy

**Figure 5:**
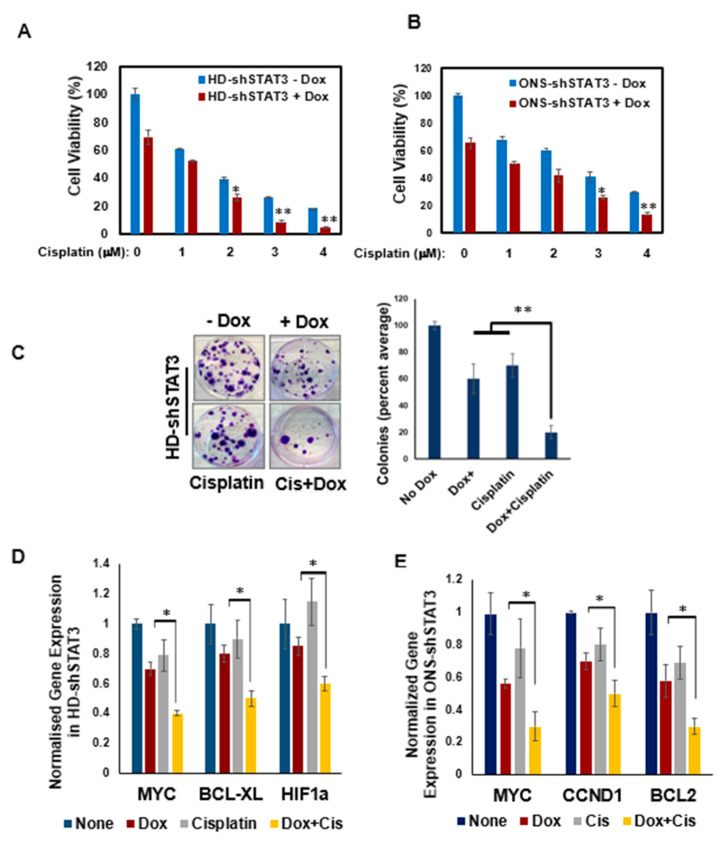

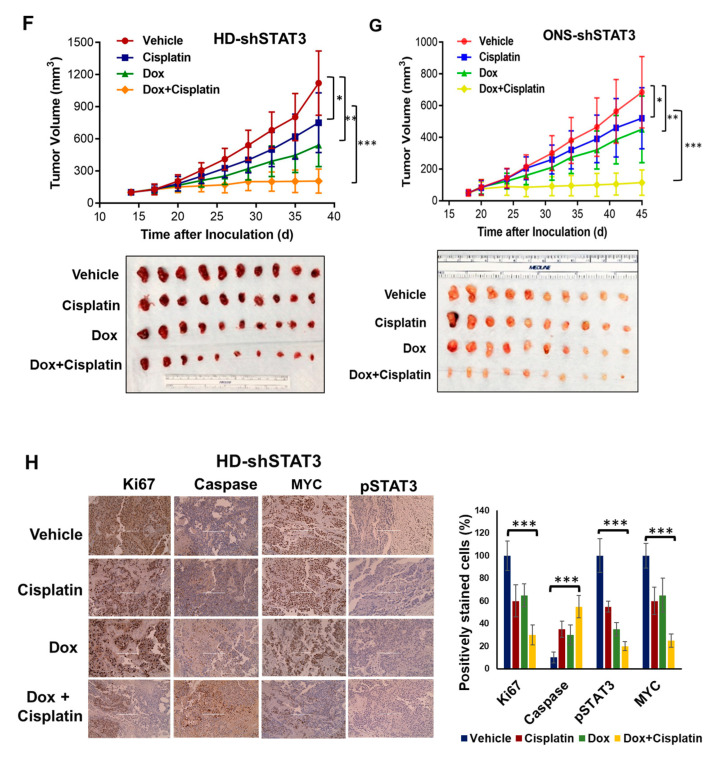
STAT3 KD confers chemosensitivity to MB cells and tumors. (**A**) HD-shSTAT3 and (**B**) ONS-shSTAT3 cells were treated either with increasing concentrations of cisplatin alone or in combination with 0.5 μg/mL Dox for 48 h and cell viability was measured by MTT assay; * *p* < 0.05, ** *p* < 0.01. (**C**) HD-shSTAT3 cells were treated with either Dox or cisplatin alone or in combination or left untreated for 12 days. Colonies formed were fixed, stained (left) and counted from triplicate wells and are shown in a bar diagram (right); ** *p* < 0.01 (**D**) STAT3 target gene expressions were analyzed in HD-shSTAT3 and (**E**) ONS-shSTAT3 cells by qPCR, either in the presence of Dox or cisplatin alone, or in combination; * *p* < 0.05. (**F**) HD-shSTAT3 and (**G**) ONS-shSTAT3 cells (0.5 × 10^6^) were subcutaneously implanted in 5- to 6-week-old athymic nude mice. Mice were given water supplemented either with Dox (2 mg/mL in drinking water) or saline (vehicle), which was continued throughout the experiment. Cisplatin (2 mg/kg) was injected twice a week intraperitoneally or in combination with Dox. The tumor size in treated groups (Dox, cisplatin and Dox + cisplatin) was compared with that of vehicle. Tumor volume was measured at indicated days and a tumor growth curve was plotted (top). Resected xenograft tumors (bottom) after completion of treatment were shown. Plotted values and error bars represent mean ± SEM. (**H**) Representative IHC images (20×) of the xenograft sections of Ki-67, caspase-3, MYC and pSTAT3 are shown. The percentage of Ki-67-, caspase-3-, MYC- and pSTAT3-positive cells derived from histology scores were quantified in the three tumor sections of each treatment group; *** *p* < 0.001 (ANOVA).

Chemoresistance remains one of the major hurdles in the treatment of MB [1,44]. We, therefore, asked if endogenous STAT3 levels regulate cisplatin responsiveness, as this drug is commonly used in therapies for high-risk MB and carries with it significant toxicities. To evaluate if STAT3 KD can enhance the efficacy of cisplatin therapy for MB, we treated HD-shSTAT3 and ONS-shSTAT3 cells with an increasing concentration of cisplatin, either alone or in combination with 0.5 μg/mL Dox. Treatment of HD-shSTAT3 and ONS-shSTAT3 cells with 3 μM cisplatin alone resulted in reduced cell viability (26% and 40%, respectively) (Figure 5A,B). However, when combined with Dox, inhibition of cell growth dropped to ∼8% and ∼25%, respectively. Further, the cell viability of HD-shSTAT3 and ONS-shSTAT3 cells with 4μM cisplatin alone decreased from 18% and 29% to 4% and 13%, respectively, with STAT3 KD. These results indicate that the combined treatment of STAT3 KD with cisplatin reduced MB cell growth significantly, with a combination index (CI) < 1, suggesting that the effect was synergistic (HDshSTAT3:CI = 0.86 and ONS-shSTAT3:CI = 0.91), and that, therefore, targeting STAT3 could be a promising strategy to sensitize chemoresistant MB cells to cisplatin [36]. Furthermore, we investigated if STAT3 KD concurrent with cisplatin affected MB colony formation and expression of downstream STAT3 target genes. Figure 5C shows that the combination of Dox and cisplatin significantly downregulated the number of colonies and attenuated gene expression of MYC, BCL-XL, HIF1a, CCND1, and BCL2 when compared to single-agent treatment (Figure 5D,E). These results indicate that the combination treatment sensitized MB cells to cisplatin and reduced the expression of STAT3 target genes, including MYC.

### 3.6. STAT3 Genetic Inhibition In Vivo Suppresses MB Tumor Growth and Sensitizes MB Tumor to Cisplatin

To examine whether STAT3 KD inhibits tumor growth in vivo and sensitizes MB tumors to cisplatin treatment, we first utilized a subcutaneous tumor xenograft model with HD-shSTAT3 and ONS-shSTAT3 cells (Figure 5F,G). Tumor growth curve analysis (top) and tumor sizes (bottom) showed that single-agent treatment, with either cisplatin (2 mg/kg twice a week; i.p.) or Dox (2 mg/mL in drinking water) alone for four weeks had a moderate effect on tumor growth compared to the vehicle group (saline). However, the combination of Dox and cisplatin significantly inhibited the xenograft growth (Figure 5F,G). IHC analysis of Ki-67, MYC, and caspase-3 (CC3) in tumor sections of HD-shSTAT3 and ONS-shSTAT3 xenografts showed that combination treatment suppressed cell proliferation and increased the number of apoptotic cells (Figure 5H and Figure S9). Consistent with the in vitro results, Dox treatment also decreased the staining intensity of pY705-STAT3 in these xenografts. Further, histologic examination of major organs, including liver, lung, spleen, heart, and kidneys, did not show any toxicity after completion of the treatment (Figure S10). Together, these data suggest that Dox augments cisplatin treatment in vivo, and the combination decreases MB tumor burden and cellular proliferation, while increasing apoptosis.

### 3.7. WP1066 Decreases MB Tumor Burden in Subcutaneous and Intracranial Orthotopic Xenograft Models and Augments MB Chemosensitivity

**Figure 6:**
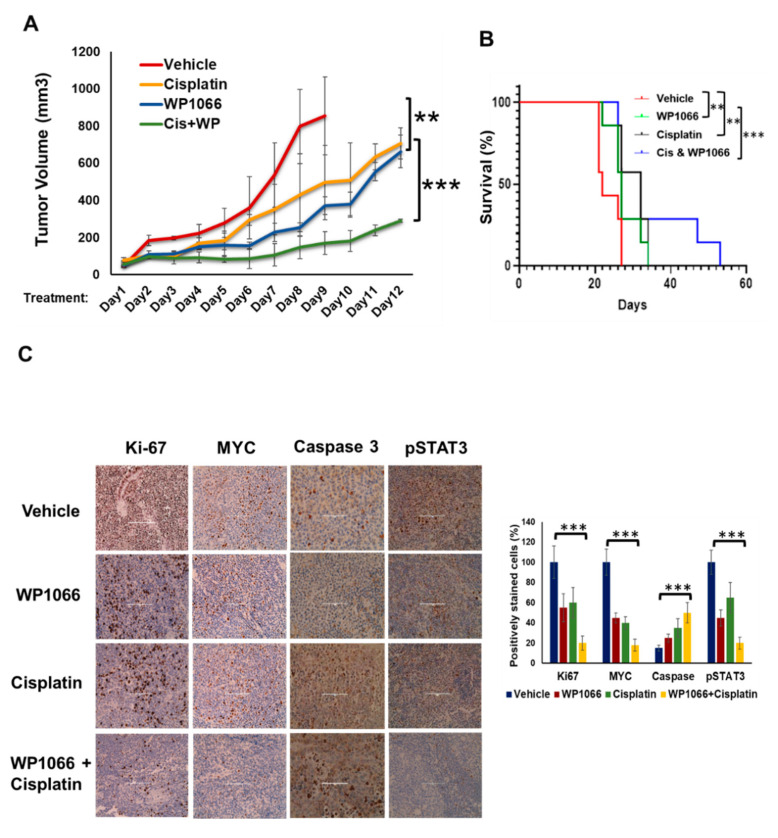

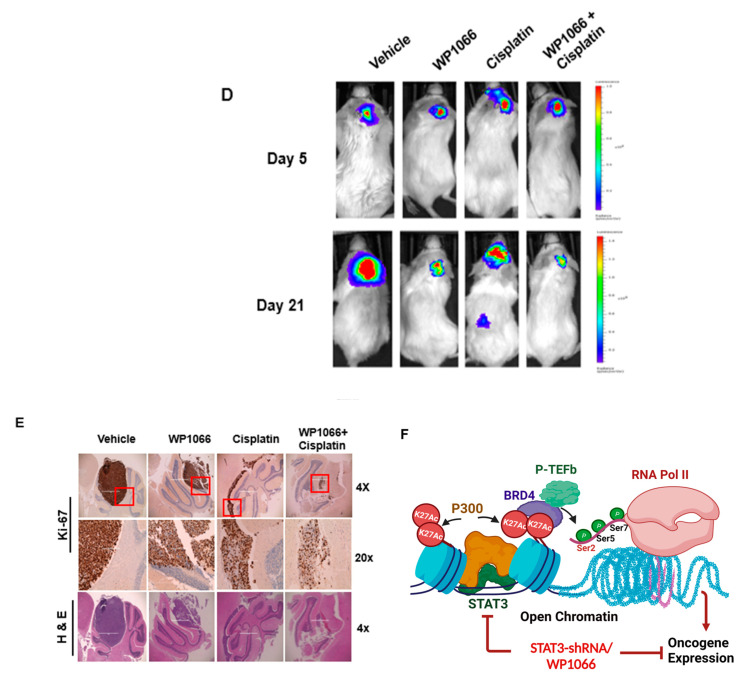
WP1066 attenuates MB tumor growth, augments chemosensitivity and mice survival. (**A**) HD-MB03 cells (0.5 × 10^6^) were subcutaneously implanted in 5- to 6-week-old athymic nude mice. When palpable tumors were reached, treatment was initiated. WP1066 (30 mg/kg) was given 3 days a week for 4 weeks and cisplatin (2 mg/kg) twice a week for 4 weeks. DMSO (5%) was given as vehicle control. Tumor volume was measured at indicated days and tumor growth curves (Vehicle, WP1066, cisplatin and WP1066 + cisplatin) were plotted. (**B**) Survival curves were plotted using a Kaplan–Meier analysis. The *p* value was calculated using a 2-sided log-rank test. (**C**) Representative IHC images (20×) of the xenograft sections of Ki-67, caspase-3, MYC and pSTAT3 are shown. The percentages of Ki-67-, caspase-3-, MYC- and pSTAT3-positive cells derived from histology scores were quantified in the three tumor sections of each treatment group and are shown in a bar diagram. (**D**) Luciferase-expressing HD-MB03 cells were implanted orthotopically into the cerebella of NSG mice for tumor growth and the mice were treated with either WP1066 or cisplatin or the combination of both. IVIS images of representative mouse from each treatment group at day 5 (treatment started), and at day 21 (mice sacrificed) after implantation are shown. (**E**) H&E staining and IHC staining of cerebellar section of mice brain show the presence of tumor and proliferating cells; ** *p* < 0.01, *** *p* < 0.001 (ANOVA). (**F**) Proposed working model by which activated STAT3 and its co-regulators promote oncogene expression and chemoresistance in MB and how it could be targeted by STAT3 inhibition.

Next, to evaluate the therapeutic efficacy of the STAT3 small molecule inhibitor WP1066, which inhibits STAT3 phosphorylation in MB cells (Figure S11) and is under pediatric clinical trial (NCT04334863), we treated subcutaneous xenograft models of HD-MB03 with WP1066 in vivo. Our results showed that, although the i.p. injection of either WP1066 (at 30 mg/kg 3 days/week) or cisplatin (2 mg/kg twice a week) could decrease the growth of subcutaneous xenograft models, the combination of WP1066 and cisplatin caused a greater reduction in tumor growth (Figure 6A). We observed that treatment with WP1066 or cisplatin alone suppressed the tumor growth by only 22% and 18%, respectively, compared to vehicle control, whereas, when WP1066 and cisplatin treatments were combined, tumor growth was reduced by 65%. In addition, after termination of the treatment at 28 days, mice receiving the combined treatment survived for another 4 weeks, suggesting the antitumor and life-prolonging potential of WP1066 and cisplatin combination treatment against MYC-driven MB in vivo (Figure 6B). The combination of WP1066 and cisplatin was well tolerated at the scheduled doses as there was no significant difference in weight loss in mice among groups (Figure S12). Histologic examination of major organs after completion of the treatment revealed no toxicity (Figure S13). IHC analyses of xenograft tumors showed that, while WP1066 and cisplatin alone reduced the expression of MYC and Ki-67 and induced the expression of CC3, the combination of WP1066 and cisplatin further significantly reduced the expression of MYC and Ki-67 and increased expression of CC3 in xenografted tumors (Figure 6C).

Since the permeability of WP1066 through the blood-brain barrier has been demonstrated in glioma models [45], we next tested its therapeutic efficacy in an orthotopic xenograft model of MYC-driven MB. HD-MB03 cells stably expressing RFP/luciferase were injected into the cerebellum of NSG mice. Five days after implantation, IVIS imaging measurements were taken to ensure the establishment of intracranial tumors. Treatment was started at day 5 (first dose), with either vehicle, WP1066 or cisplatin, or the combination of both, every other day for 16 days, as stated in the Materials and Methods section. IVIS imaging measurements were taken at day 21, then mice were sacrificed, and brains were harvested. Although cisplatin and WP1066 treatment alone reduced the tumor growth, the combination of both substantially suppressed intracranial growth of MB tumors (Figure 6D). Further, H&E staining and IHC analysis of Ki67 revealed a much larger tumor at the cerebellum region of the vehicle-treated mouse compared to the WP1066 or cisplatin treatment (Figure 6E). Further, a significant suppression of tumor growth was observed in WP1066-plus-cisplatin-treated mice, suggesting that the combination of WP1066 and cisplatin effectively suppressed MB tumor growth in an orthotopic xenograft model.

## 4. Discussion

Persistently activated STAT3 is often implicated in tumorigenesis of multiple malignancies, including MB [46,47]. Our laboratory and others showed that increased STAT3 phosphorylation, which occurs downstream of Janus-activated kinases (JAK), is commonly associated with expression of pro-survival and oncogenic proteins that leads to MB pathogenesis. A recent study showed that autocrine interleukin-6/STAT3 signaling promotes development of acquired drug resistance in the most aggressive Group 3 MB and a previous study demonstrated that the STAT3 pathway is selectively activated in CD133+ MB stem cells and promotes MB tumorigenesis; however, the underlying molecular mechanisms were not determined [22,48]. Moreover, some emerging evidence has demonstrated that perturbing STAT3 function in MB inhibits cell and tumor growth; however, a detailed mechanistic study using a systematic approach, considering the inducible genetic KD and simultaneous effect of pharmacologic inhibition of STAT3 on MYC gene regulation, orthotopic MB tumor growth and chemosensitivity, has not been performed in MB [14,48,49,50]. In this study, by cross-validating cellular phenotypes using both shRNA knockdown of STAT3 and a clinically relevant STAT3 inhibitor WP1066, we have established a key role of STAT3 in promoting MB tumorigenesis and chemoresistance and activation of the MYC oncogene. We provide multiple lines of evidence that STAT3 inhibition impacts tumorigenic attributes in MB cells, including cell survival, proliferation, apoptosis, migration and stemness in vitro. It also suppressed MB tumor growth in both subcutaneous and intracranial orthotopic group 3 MB xenografts in vivo. We elucidated the molecular basis by which STAT3 inhibition reduced expression of the key oncogene MYC [37]. Our findings suggest that STAT3 knockdown reduced recruitment of histone acetyltransferase p300 and subsequently reduced levels of histone H3K27 acetylation on MYC gene promoter/enhancer regions, resulting in reduced occupancy of BRD4 and RNA Pol II loading. Further, we demonstrated that KD or inhibition of STAT3 with WP1066 not only suppressed MB tumor growth, but also augmented the sensitivity of MB tumors to cisplatin. Of note, WP1066 is currently in phase 1 pediatric cancer clinical trials [NCT04334863] and has the ability to cross the blood–brain barrier, which is clinically imperative [45]. 

Although MYC gene overexpression/and or amplification is often observed in group 3 MB patients, the key regulatory factors that precisely control MYC expression in MB are still unclear. Detailed classification within group 3 MB by Cavalli et al. reported that group 3γ has high MYC amplification, high rates of metastasis, and worse overall survival [51]. Coincidently, increased levels of both IL-6 and STAT3 have been reported in group 3 MB compared to an SHH group; more specifically STAT3 levels in groups 3β and 3γ were significantly higher than other groups, correlating with the severity of outcomes [22]. As direct targeting of MYC in cancer thus far has been unsuccessful because of its complex structure and pleiotropic functions, the identification of key cofactors for MYC to target at transcriptional or translational levels has emerged as a promising alternative for the indirect inhibition of MYC [52,53]. Our study identified STAT3 as a key regulator for the expression of proto-oncogene MYC in MB cells and tumors. It has been reported that 17% of all activated genes in tumors are regulated by the transcription factor MYC [54]. Consistent with this, our RNA-seq analysis and gene ontology analysis of differentially expressed genes further highlighted that MYC target genes and MYC-driven pathways are among the top biological processes affected by STAT3 KD.

STAT3-driven transcription requires several cofactors (such as CBP/p300, BRD4, NcoA/SRC1a, PTEF-b, etc.). These cofactors induce modifications on histone tails or non-histone proteins, coupled with chromatin remodeling to activate the basal transcription machinery and enhance gene expression [26,27,55,56,57]. Consistent with these findings, we propose that activated STAT3 binds and recruits transcriptional co-activator p300 to its target gene promoters, which acetylates histone tails. Subsequently, BRD4, which features bromodomains that recognize the side-chains of acetylated histone H3, aids the recruitment of the positive transcription elongation complex b (P-TEFb) to phosphorylate serine 2 of the RNA pol II C-terminal domain to facilitate transcription elongation and gene activation (Figure 6F). Further study by ChIP-seq analysis is warranted to examine the impact of STAT3 inhibition on genome-wide alteration of H3K27Ac enhancer marks at promoter/enhancer regions in MB cells.

A recent interesting finding in mouse models of glioma by Sweha et.al. showed that histone H3.3G34 mutations epigenetically activate leukemia inhibitory factor (LIF) by promoter hypomethylation and enrichment of activation mark H3K27Ac. Increased secretion of LIF in turn activates the STAT3 signaling pathway and, therefore, WP1066 was able to suppress tumor growth and greatly improve mouse survival. This suggests that epigenetic reprogramming of target genes by histone modification plays an important role in pediatric cancers where the mutation burden is low compared to adult cancers [45].

Although current multimodal approaches in MB have significantly improved overall survival rates, high doses of cytotoxic chemotherapies are often associated with long-term toxicities, including neurocognitive impairments and ototoxicity in surviving patients [58,59]. Moreover, among the four major subgroups of MB, tumor recurrence due to survival of therapy-resistant cells in the residual tumor is more frequently observed in SHH MB and MYC-driven group 3 MB patients and, therefore, remains a major clinical challenge for MB treatment. In this regard, the tumor-initiating cancer stem-like cells, which are thought to be resistant to therapy, have the ability to form a secondary tumor at relapse [60,61]. STAT3 is known to play an important role in the maintenance of cancer-stem-cell-like subpopulations in many cancer types [62,63]. Our data provide evidence that a subset of MB tumor-initiating cells have the ability to form tumor-spheres in serum-free conditions in vitro, which is dependent on STAT3 expression. Further, while we provide evidence that stem-cell-like marker expressions (CD133, SOX2, OCT3/4) are inhibited by STAT3 KD in these cells, it is beyond the scope of our present study to further investigate the phenotypic nature of these tumor-initiating cells or to test whether inhibition of STAT3 can prevent tumor recurrence in vivo. 

Our study demonstrated that either KD, or inhibition of STAT3 with small molecule inhibitor WP1066, significantly increases apoptosis and suppresses subcutaneous MB tumor growth with a concomitant increase in the survival of tumor-bearing mice in vivo. Genetic inhibition of STAT3 by shRNA would perturb STAT3 biological activities mediated by both unphosphorylated and phosphorylated STAT3. However, our observation that both genetic and pharmacologic inhibition of STAT3 yielded similar results of tumor growth inhibition indicates that the biological effects are direct results of STAT3 inhibition in the tumor. Consistent with this, we showed that treatment with WP1066 significantly reduced phosphorylated STAT3 levels in MB xenograft tissue section, and our previous study demonstrated that STAT3 is constitutively activated in MB patients’ tissues [14], further supporting our in vivo observations in mice. Of note, our findings were that WP1066 not only suppresses high-risk HD-MB03 tumor growth, but also increases the sensitivity of MB to cisplatin in an intracranial orthotopic model. This suggests that STAT3 inhibition, in combination with conventional cytotoxic cisplatin therapy, is a promising strategy to improve the effectiveness of chemotherapy against therapy-resistant MB. Although multiple factors promote resistance to therapy, our in vivo evidence supports the view that activation of STAT3 and overexpression of MYC in group 3 MB are linked with MB tumorigenesis and chemoresistance. Further, investigational parameters to test our findings using patient derived xenografts (PDX) in an MB orthotopic model are currently under development in our laboratory. Overall, this study suggests the potential for molecularly targeted therapies in patients with high-risk MB to increase the efficacy of conventional chemotherapy and decrease the morbidities and toxicities associated with high doses of cisplatin-based chemotherapy. Therefore, future preclinical/clinical studies in MB with selective STAT3 inhibitors in combination with cisplatin are warranted.

## 5. Conclusions

In this study, using inducible STAT3 shRNA and a clinically relevant STAT3 inhibitor WP1066, we have unraveled the mechanism by which STAT3 induces expression of the driver oncogene MYC and promotes MB tumorigenesis and chemoresistance. Of note, we identified that a STAT3 inhibitor augments the efficacy of standard chemotherapy and inhibits MB tumor growth in an intracranial orthotopic mouse model. Together, the study results provide proof-of-principle and a foundation for future studies to test the potential of STAT3 inhibitors as an adjuvant therapy for the treatment of high-risk MB patients, to reduce chemotherapy-related side-effects, and improve the quality of life of pediatric patients. 

## Data Availability

The data generated and/or analyzed during this study are available within the article and from the corresponding author on reasonable request.

## Supplementary Materials

The following supporting information can be downloaded at: https://www.mdpi.com/article/10.3390/cancers15082239/s1. Figure S1: Expression of red fluorescence protein (RFP) in HD-shSTAT3 (shRNA-ID-376016 and 376017) and ONS-shSTAT3 (shRNA-ID-376016) cells after 24 h of Dox treatment. Figure S2: Expression of STAT3 mRNA in HD-shSTAT3 (shRNA-ID-376016 and 376017) and ONS-shSTAT3 (shRNA-ID-376016) cells with 0.5 ug/mL Dox treatments are shown by qPCR (A) and Western Blot (B). Figure S3: Expression of STAT3 and pY705-STAT3 levels in HD-shSTAT3 and ONS-shSTAT3 cells after Dox treatment for 48 h. Figure S4: Top: Expression of STAT3 levels in parental HD-MB03 and ONS76 cells after Dox treatment for 48 h. Bottom: Left: Expression of STAT3 and pY705-STAT3 levels in HD-MB03 shRNA-Non-targeting cells (HD-shNTC) after Dox treatment for 48 h. Right: Gene expression of STAT3, MYC and BCL2 in HD-shNTC cells with Dox treatment for 48 h. Fold change normalized to GAPDH. Figure S5. Heat map showing significantly upregulated/downregulat ed genes from RNA Seq analysis of HD-shSTAT3 and ONS-shSTAT3 cells after STAT3 KD. Figure S6: GSE analysis for the changes in STAT3 target gene sets after STAT3 KD. FDR q < 0.25 considered significant. Figure S7: The growth of parental HD-MB03 and ONS76 cells and HD-Non targeting shRNA control cells (HD-shNTC) after Dox treatment for 48 h were determined using MTT assays. Figure S8: Left: Representative Cell cycle analysis plot using Propidium-iodide (PI) staining of HD-shSTAT3 and ONS-shSTAT3 cells after STAT3 KD. Right: Bar graph represent mean±SD and are based on three independent experiments. Statistical significance was determined using Student’s *t*-test (*p* < 0.05). Figure S9: Representative IHC images (20×) of the ONS-shSTAT3 xenograft sections of Ki-67, caspase-3, and pSTAT3 are shown. Figure S10: H&E staining of heart, lung, liver, kidney and spleen of HD-shSTAT3 and ONS-shSTAT3 xenograft sections showing no sign of toxicity after completion of the treatment. Figure S11: (A) HD-MB03 cells were treated with increasing dose of WP1066 for 6 h and cells were stimulated with IL-6 for 20 min before harvesting cells. Western immunoblot was performed with WCE and expression levels of pY705-STAT3, total STAT3, MYC and cleaved PARP were shown. (B) HD-MB03 cells were treated with indicated doses of WP1066 and MTT assay was performed to determine MB cell viability. (C) HD-MB03 cells were treated with indicated doses of WP1066 and annexin V staining was done. Representative bar graph shows percentage of apoptotic cell death after treatments. Figure S12 shows the line graph of the mean body weight of mice following treatment. Figure S13 shows the histopathology (H&E) of the vital organs of MB xenografts following 4 weeks post treatment with inhibitors. The images were scanned and captured using digital scanner EVOS Image system at 20× magnification. File S1: Original Images for Blots.
