# Supplementary material for: STAT3 Inhibition Attenuates MYC Expression by Modulating Co-Activator Recruitment and Suppresses Medulloblastoma Tumor Growth by Augmenting Cisplatin Efficacy In Vivo
Source: Cancers (Basel). 2023 Apr 11;15(8):2239. doi: 10.3390/cancers15082239 (PMC10136921; doi:10.3390/cancers15082239)
Supplement: Supplementary file 1 [file cancers-15-02239-s001.zip › cancers-2267642-supplementary.pdf]

Supplement Figures/legends

Fig. S1-S13

Fig. S1

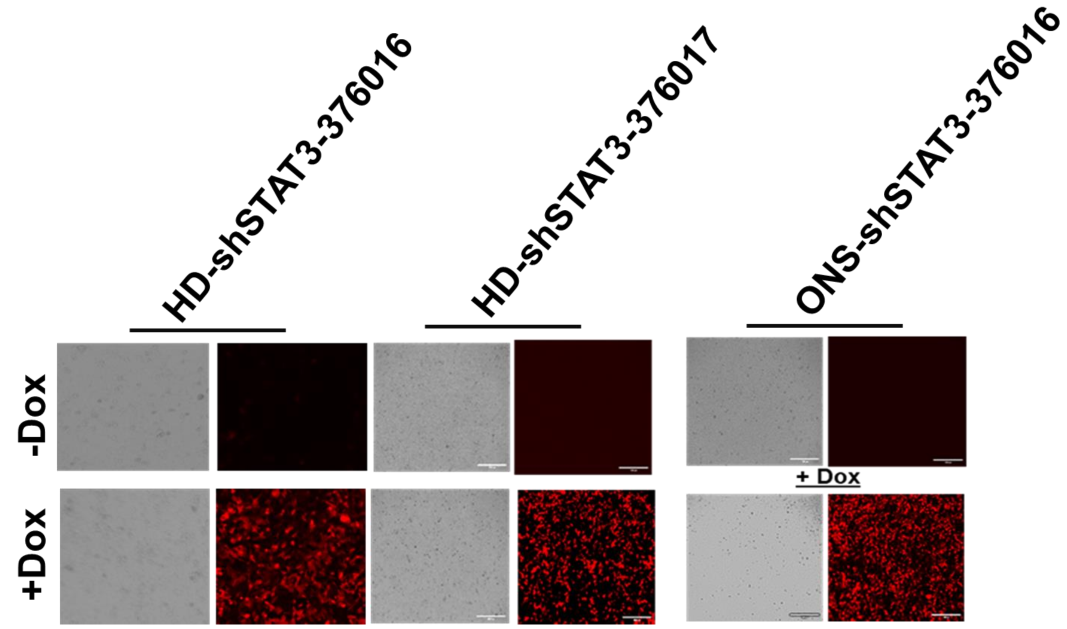

Figure S1: Expression of red fluorescence protein (RFP) in HD-shSTAT3 (shRNA-ID-376016 and 376017) and ONS-shSTAT3 (shRNA-ID-376016) cells after 24 h of Dox treatment.

Fig. S2

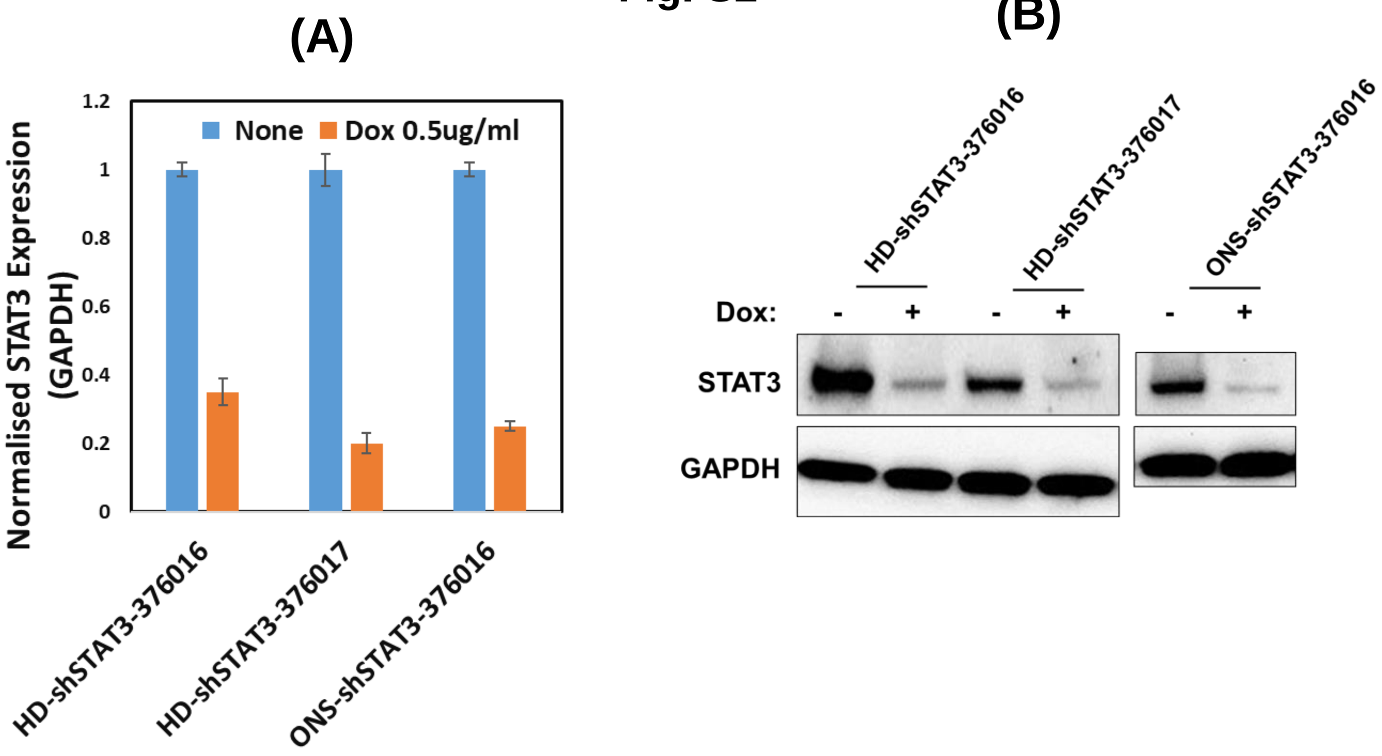

Figure S2: Expression of STAT3 mRNA in HD-shSTAT3 (shRNA-ID-376016 and 376017) and ONS-shSTAT3 (shRNA-ID-376016) cells with 0.5 ug/ml Dox treatments are shown by qPCR (A) and Western Blot (B).

Fig. S3

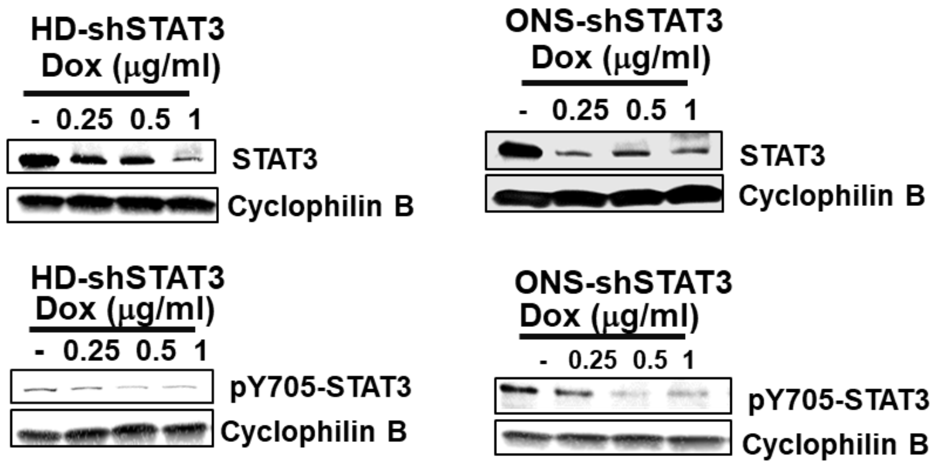

Figure S3: Expression of STAT3 and pY705-STAT3 levels in HD-shSTAT3 and ONS-shSTAT3 cells after Dox treatment for 48 h.

Fig. S4

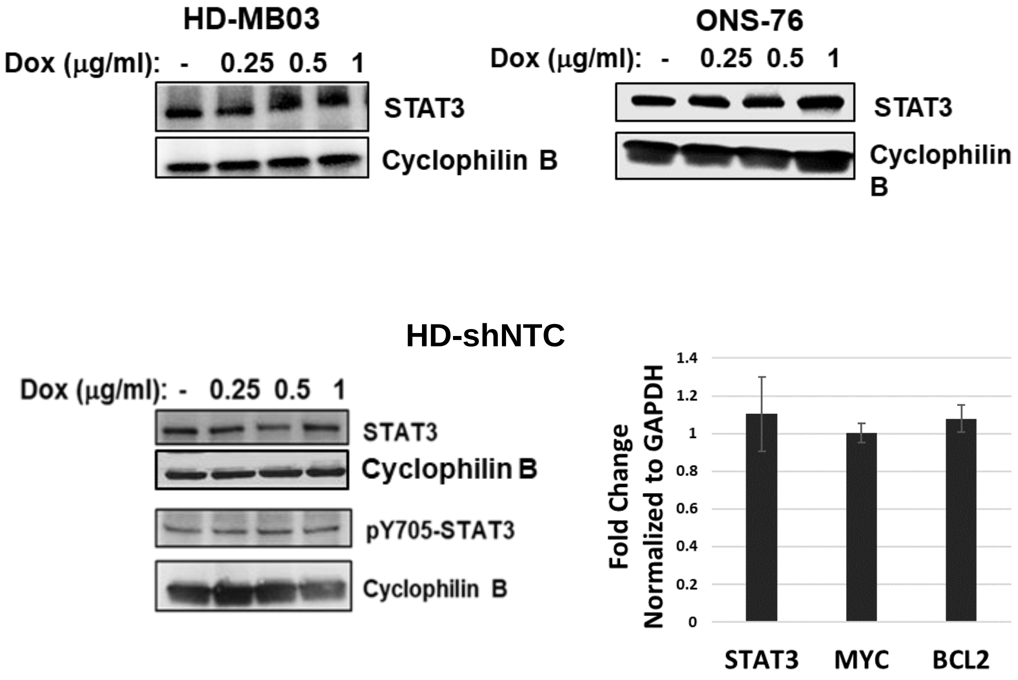

Figure S4: Top: Expression of STAT3 levels in parental HD-MB03 and ONS76 cells after Dox treatment for 48 h.

Bottom: Left: Expression of STAT3 and pY705-STAT3 levels in HD-MB03 shRNA-Non-targeting cells (HD-shNTC) after Dox treatment for 48 h. Right: Gene expression of STAT3, MYC and BCL2 in HD-shNTC cells with Dox treatment for 48 h. Fold change normalized to GAPDH.

Fig. S5

HD-shSTAT3 +/- Dox

ONS-shSTAT3 +/- Dox

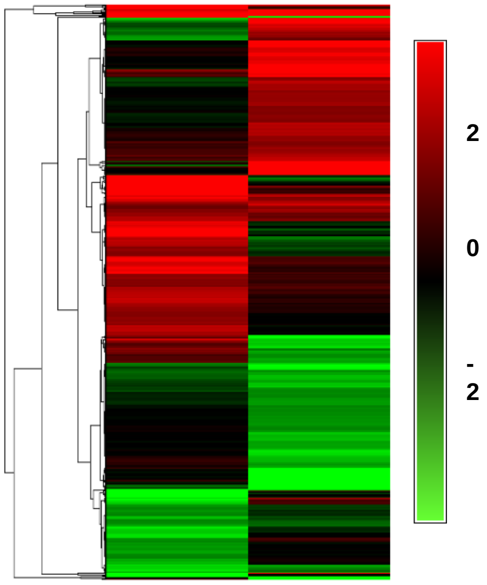

Euclidean cluster analysis of genes where the gene had a significant (FDR≤0.05) and log2 fold change ≥ 1 or ≤ -1 from control to treatment in a minimum of one comparison

Figure S5. Heat map showing significantly upregulated/ downregulat ed genes from RNA Seq analysis of HD-shSTAT3 and ONS-shSTAT3 cells after STAT3 KD.

Fig. S6

GSEA analysis of HD-shSTAT3

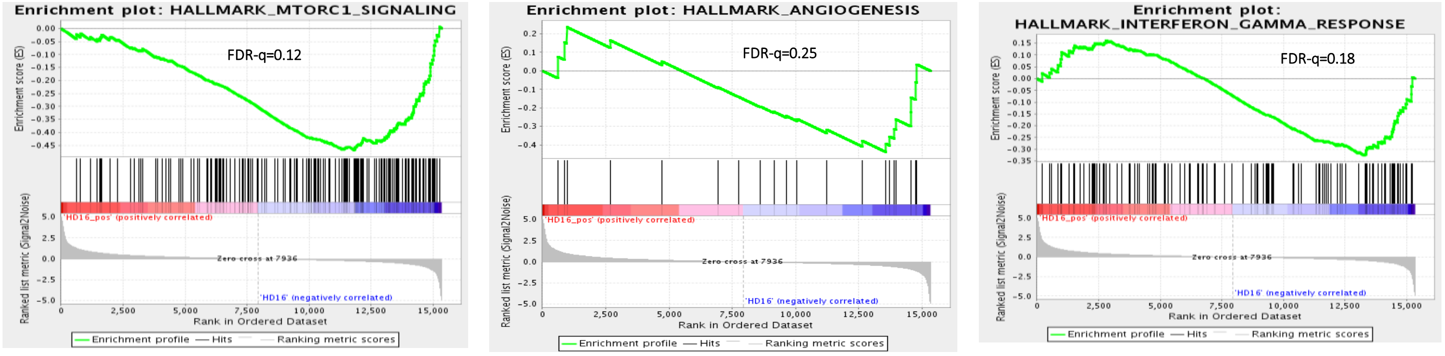

GSEA analysis of ONS-shSTAT3

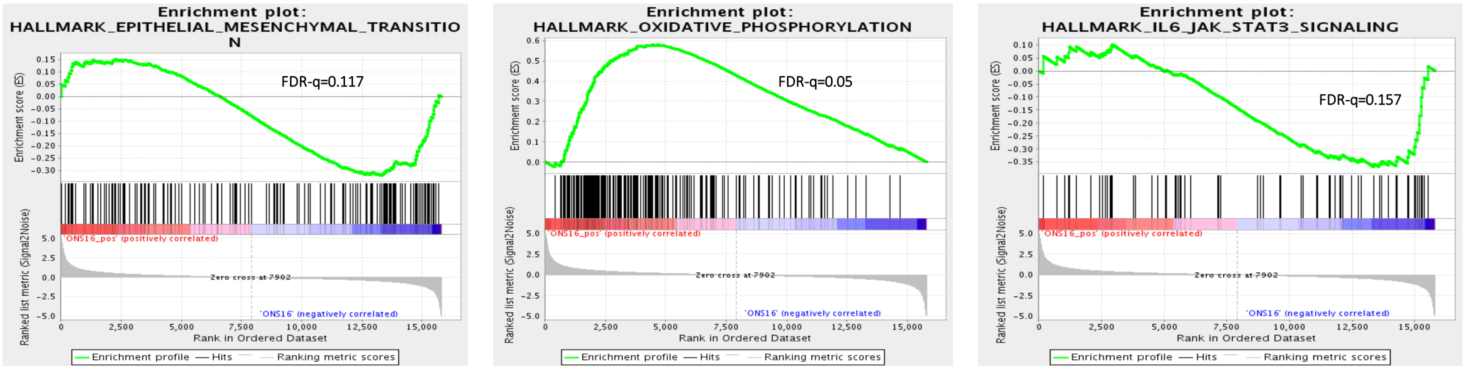

Figure S6: GSE analysis for the changes in STAT3 target gene sets after STAT3 KD. FDR q<0.25 considered significant.

Fig. S7

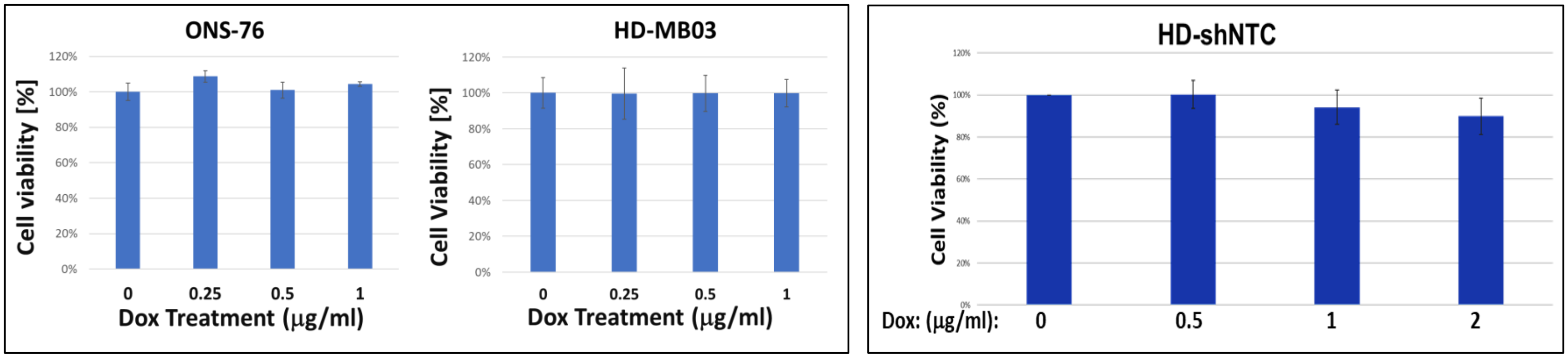

Figure S7: The growth of parental HD-MB03 and ONS76 cells and HD-Non targeting shRNA control cells (HD-shNTC) after Dox treatment for 48 h were determined using MTT assays.

Fig. S8

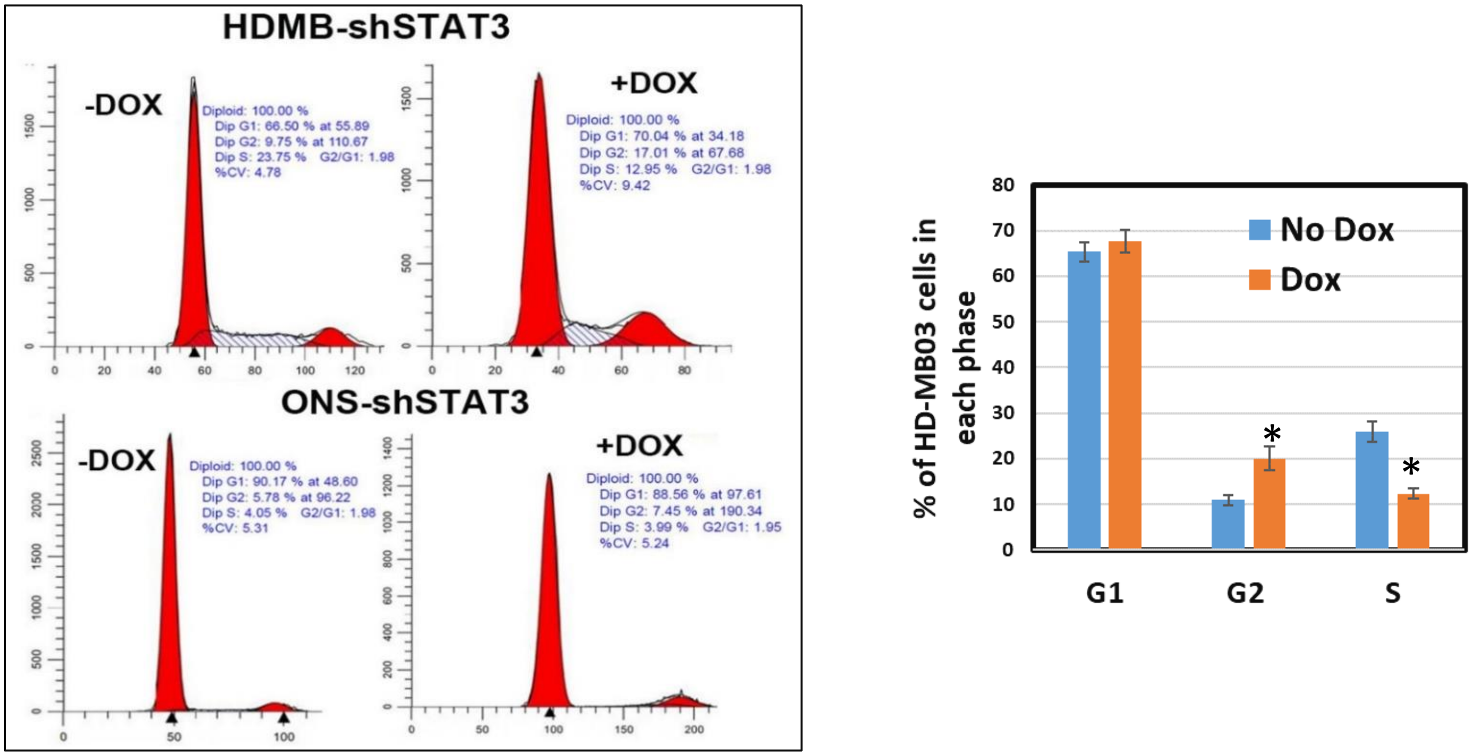

Figure S8: Left: Representative Cell cycle analysis plot using Propidium-iodide (PI) staining of HD-shSTAT3 and ONS-shSTAT3 cells after STAT3 KD. Right: Bar graph represent mean±SD and are based on three independent experiments. Statistical significance was determined using Student's t-test ($p<0.05$)

Fig. S9

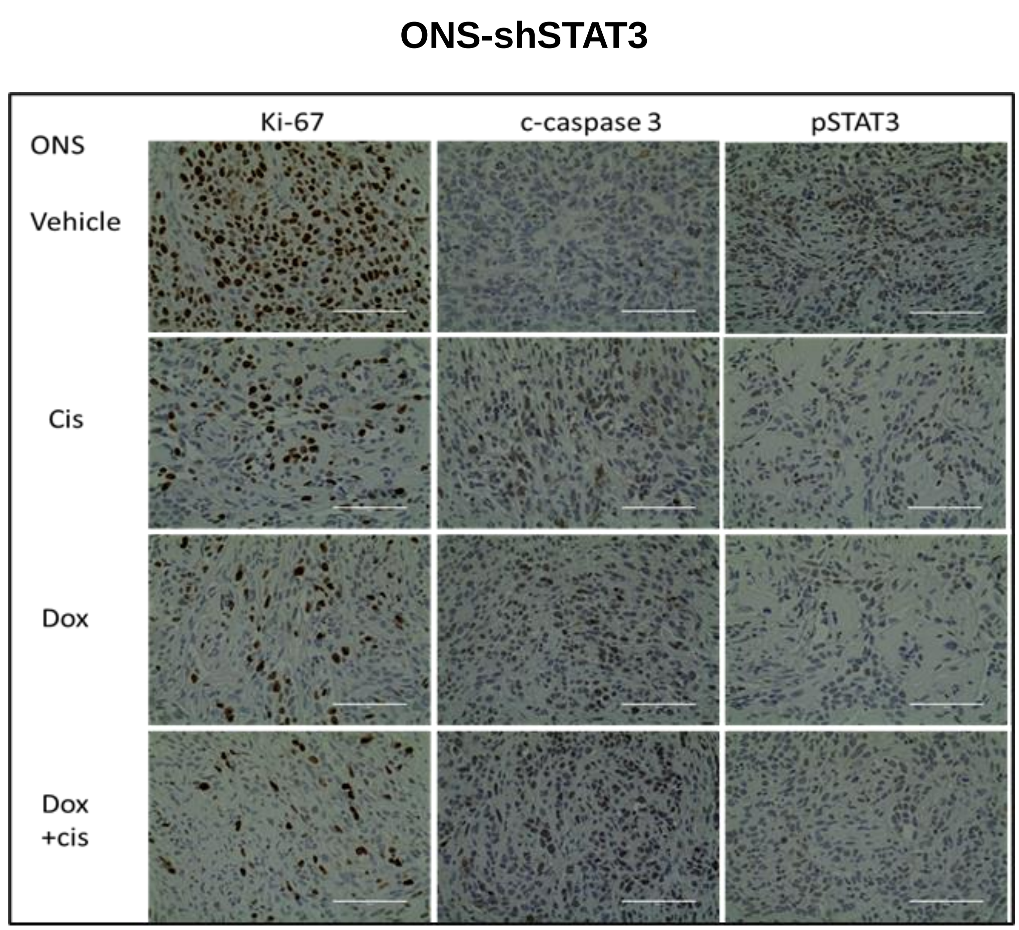

Figure S9: Representative IHC images (20X) of the ONS-shSTAT3 xenograft sections of Ki-67, caspase-3, and pSTAT3 are shown.

Fig. S10

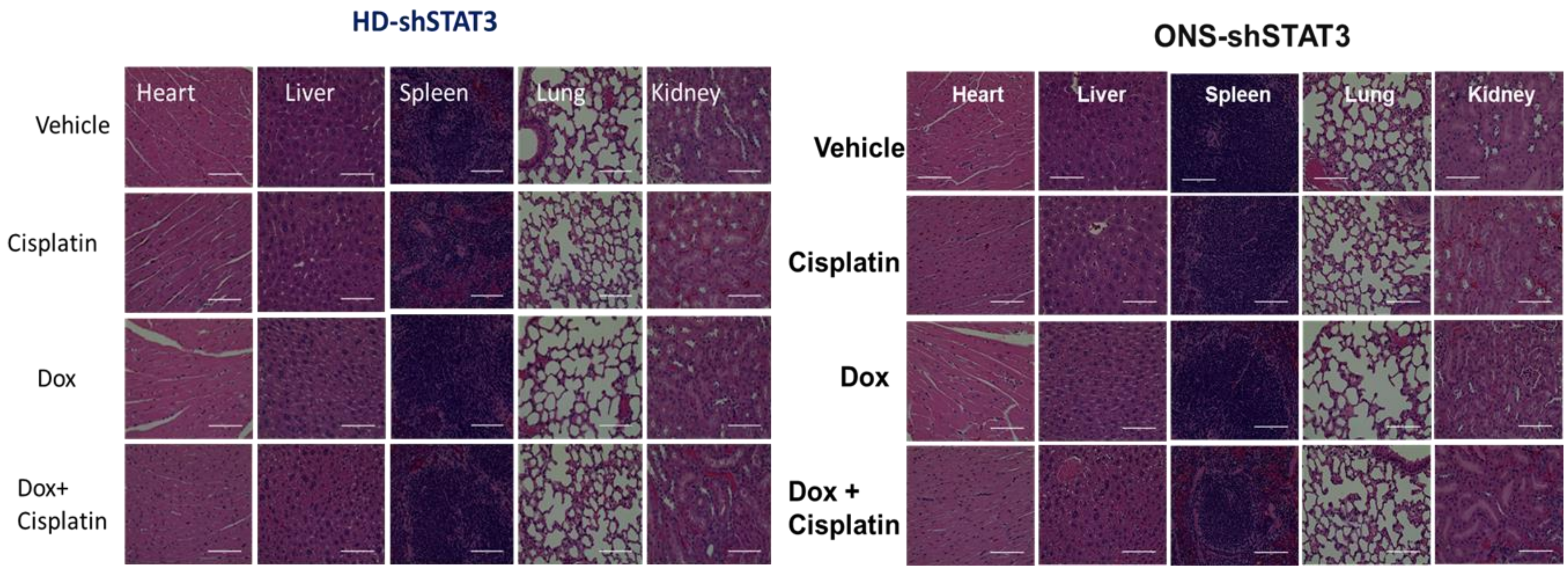

Figure S10: H&E staining of heart, lung, liver, kidney and spleen of HD-shSTAT3 and ONS-shSTAT3 xenograft sections showing no sign of toxicity after completion of the treatment.

Fig. S11

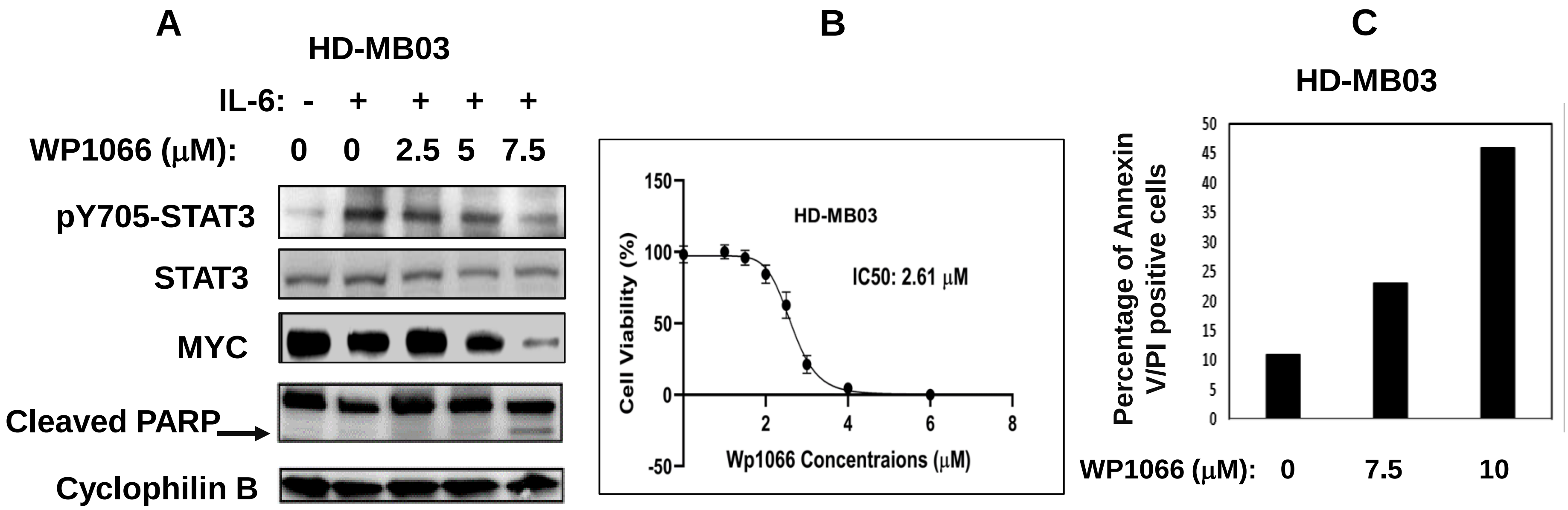

Figure S11: **(A)** HD-MB03 cells were treated with increasing dose of WP1066 for 6 hours and cells were stimulated with IL-6 for 20 minutes before harvesting cells. Western immunoblot was performed with WCE and expression levels of pY705-STAT3, total STAT3, MYC and cleaved PARP were shown. **(B)** HD-MB03 cells were treated with indicated doses of WP1066 and MTT assay was performed to determine MB cell viability. **(C)** HD-MB03 cells were treated with indicated doses of WP1066 and annexin V staining was done. Representative bar graph shows percentage of apoptotic cell death after treatments.

Fig. S12

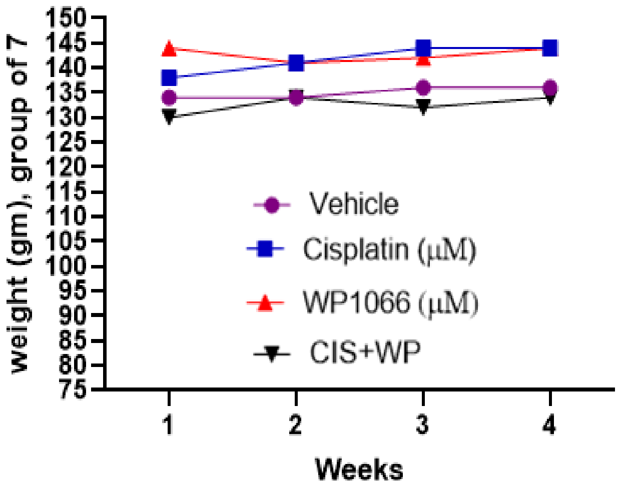

Fig. S13

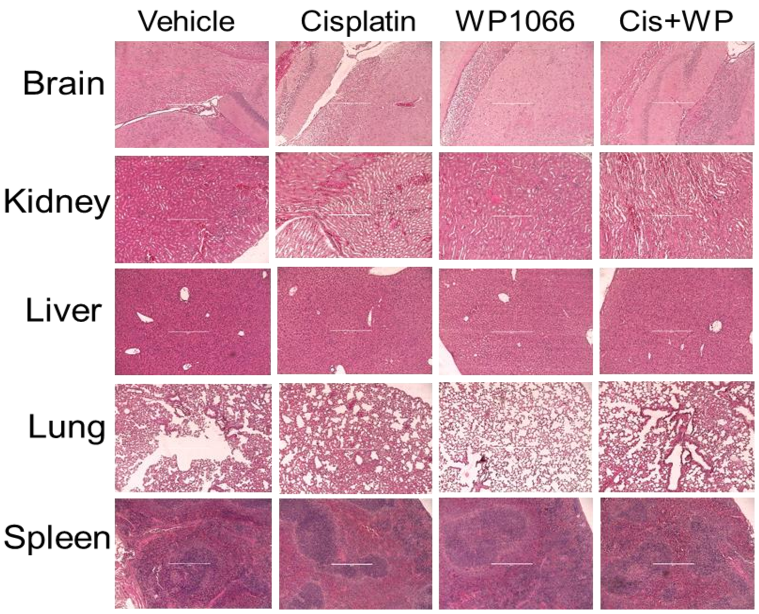

Figure S12 shows the line graph of the mean body weight of mice following treatment.
Figure S13 shows the histopathology (H&E) of the vital organs of MB xenografts following 4 weeks post treatment with inhibitors. The images were scanned and captured using digital scanner EVOS Image system at 20x magnification.
