# Supplementary material for: STAT3 Inhibition Attenuates MYC Expression by Modulating Co-Activator Recruitment and Suppresses Medulloblastoma Tumor Growth by Augmenting Cisplatin Efficacy In Vivo
Source: Cancers (Basel). 2023 Apr 11;15(8):2239. doi: 10.3390/cancers15082239 (PMC10136921; doi:10.3390/cancers15082239)
Supplement: Supplementary file 1 [file cancers-15-02239-s001.zip › File S1-Original Images for Blots.pptx]

## Slide 1
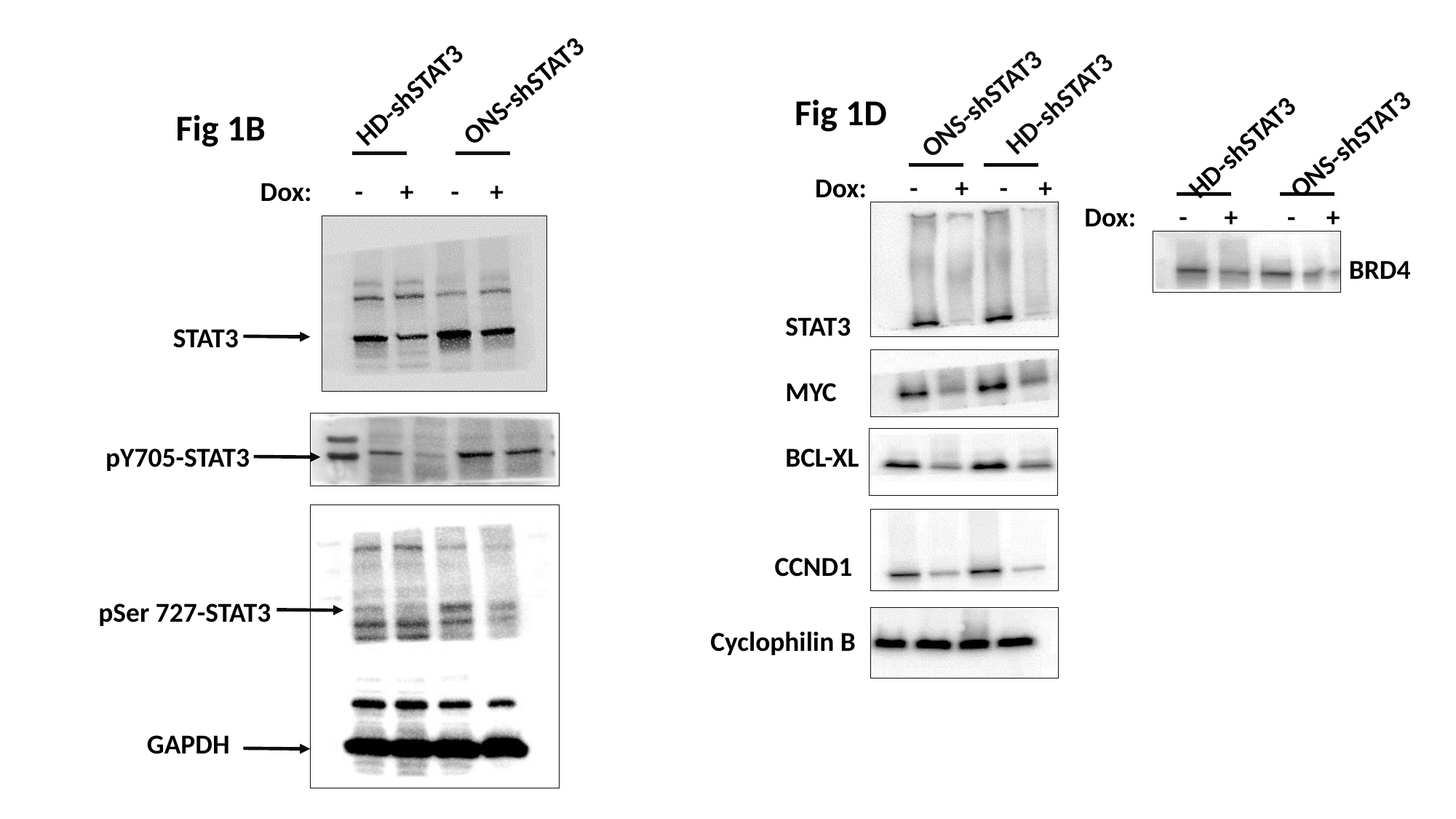

ONS-shSTAT3
HD-shSTAT3
Fig 1B
Dox: - + - +
STAT3
pY705-STAT3
pSer 727-STAT3
ONS-shSTAT3
HD-shSTAT3
Fig 1D
ONS-shSTAT3
HD-shSTAT3
Dox: - + - +
Dox: - + - +
BRD4
STAT3
MYC
BCL-XL
CCND1
Cyclophilin B
GAPDH

## Slide 2
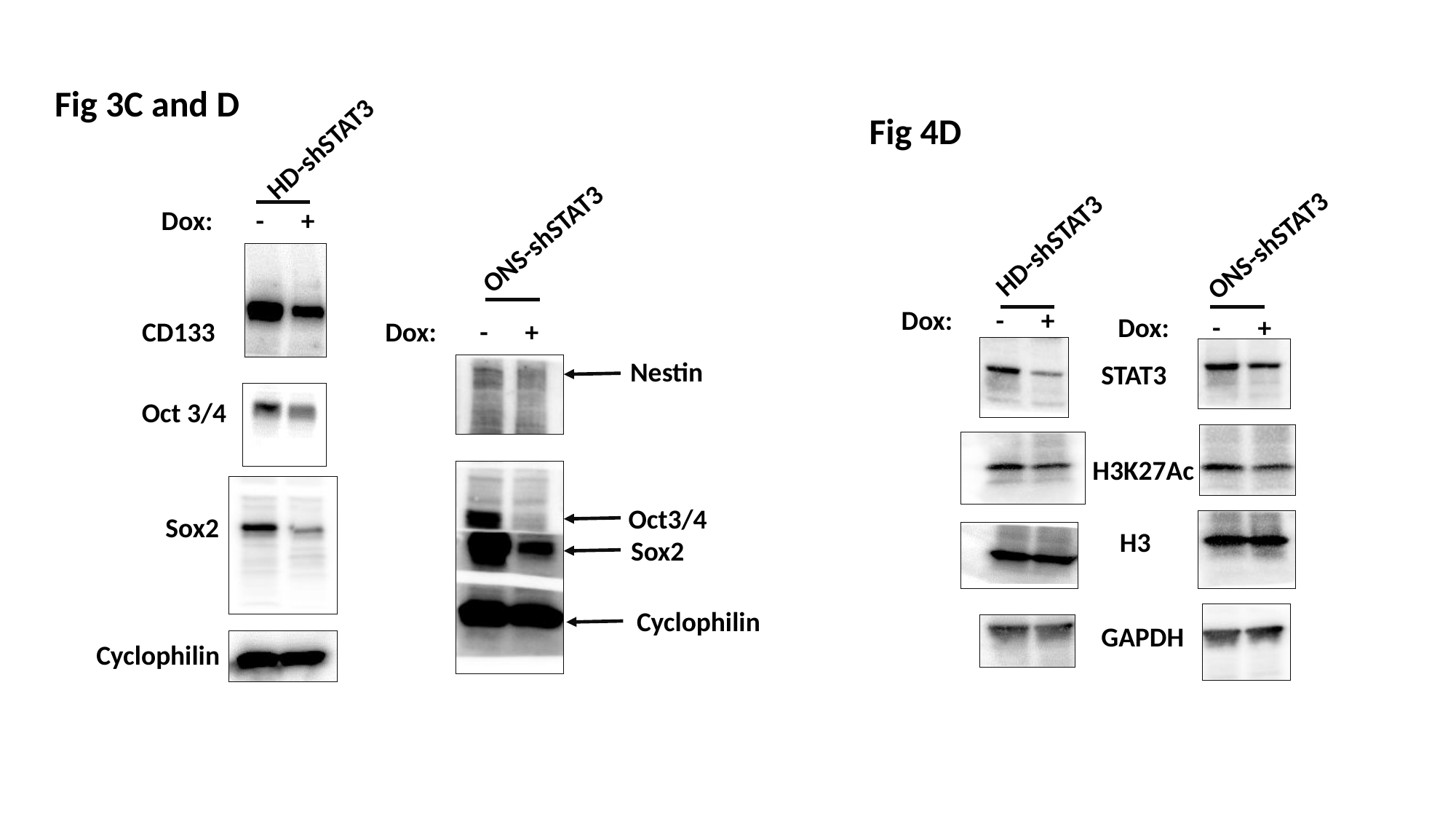

Fig 3C and D
HD-shSTAT3
Dox: - +
ONS-shSTAT3
Dox: - +
Nestin
Oct3/4
Sox2
Cyclophilin
CD133
Oct 3/4
Sox2
Cyclophilin
Fig 4D
HD-shSTAT3
ONS-shSTAT3
Dox: - +
Dox: - +
STAT3
H3K27Ac
H3
GAPDH

## Slide 3
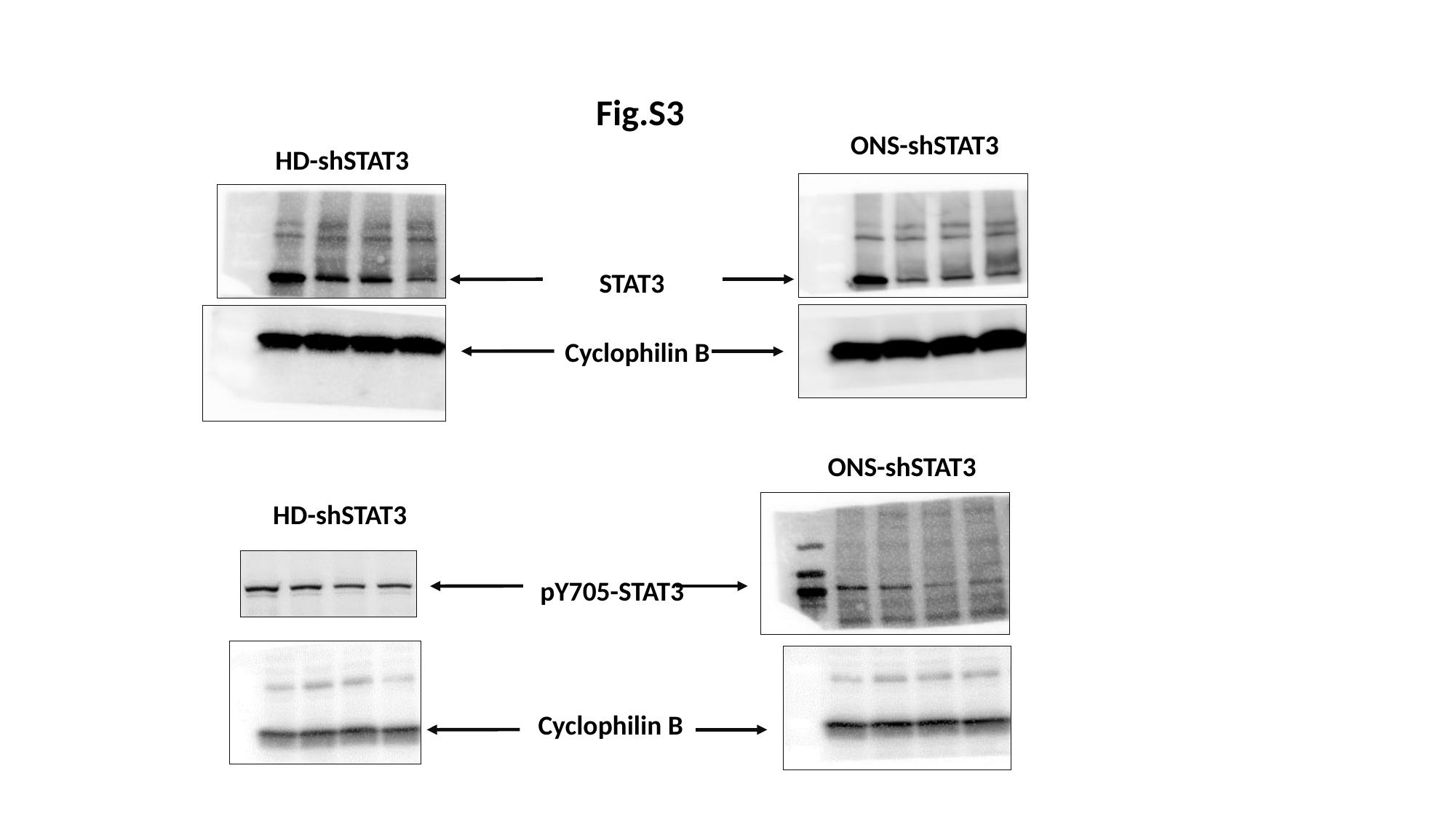

Fig.S3
ONS-shSTAT3
HD-shSTAT3
STAT3
Cyclophilin B
ONS-shSTAT3
HD-shSTAT3
pY705-STAT3
Cyclophilin B

## Slide 4
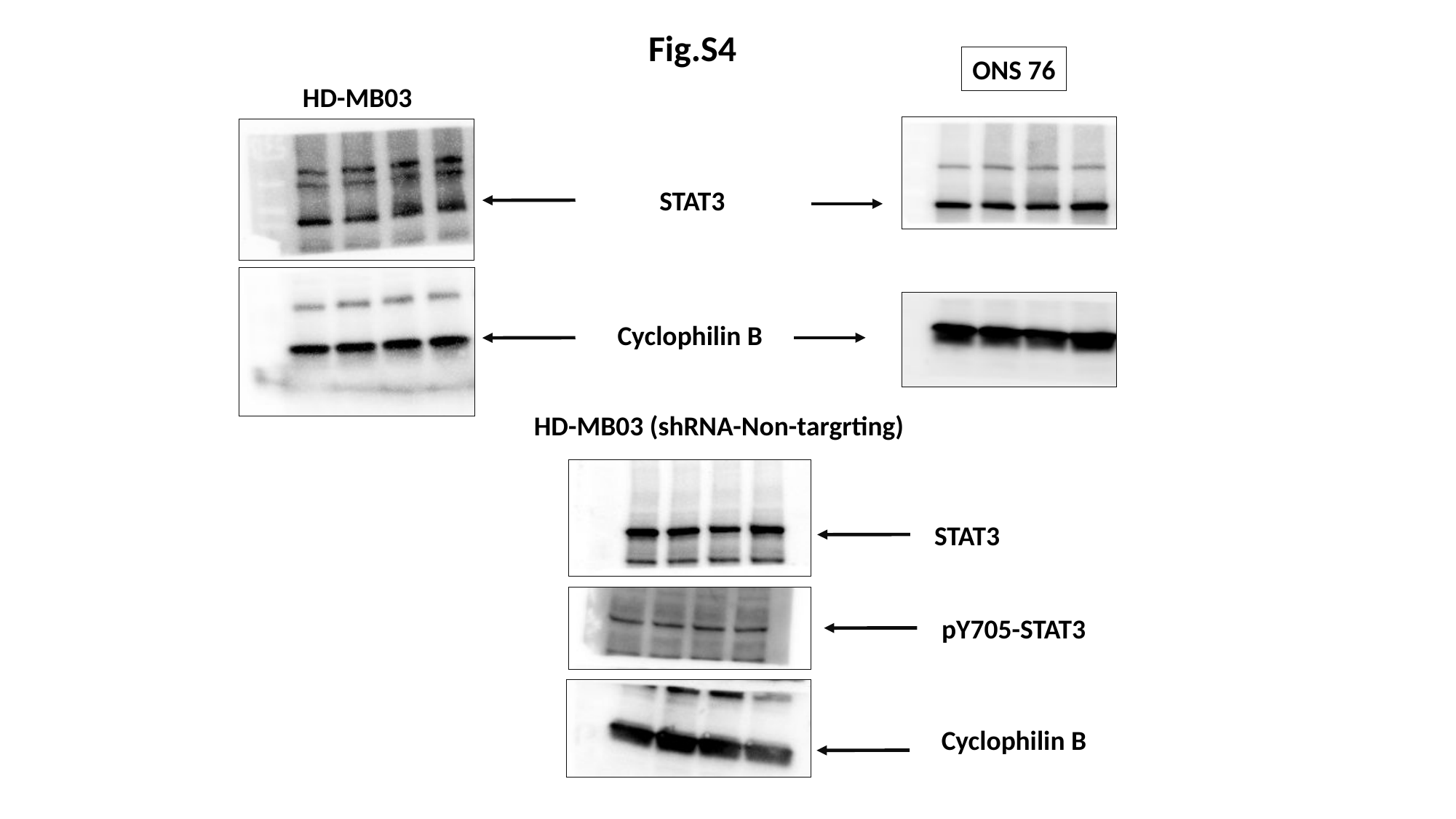

Fig.S4
ONS 76
HD-MB03
STAT3
Cyclophilin B
HD-MB03 (shRNA-Non-targrting)
STAT3
pY705-STAT3
Cyclophilin B

## Slide 5
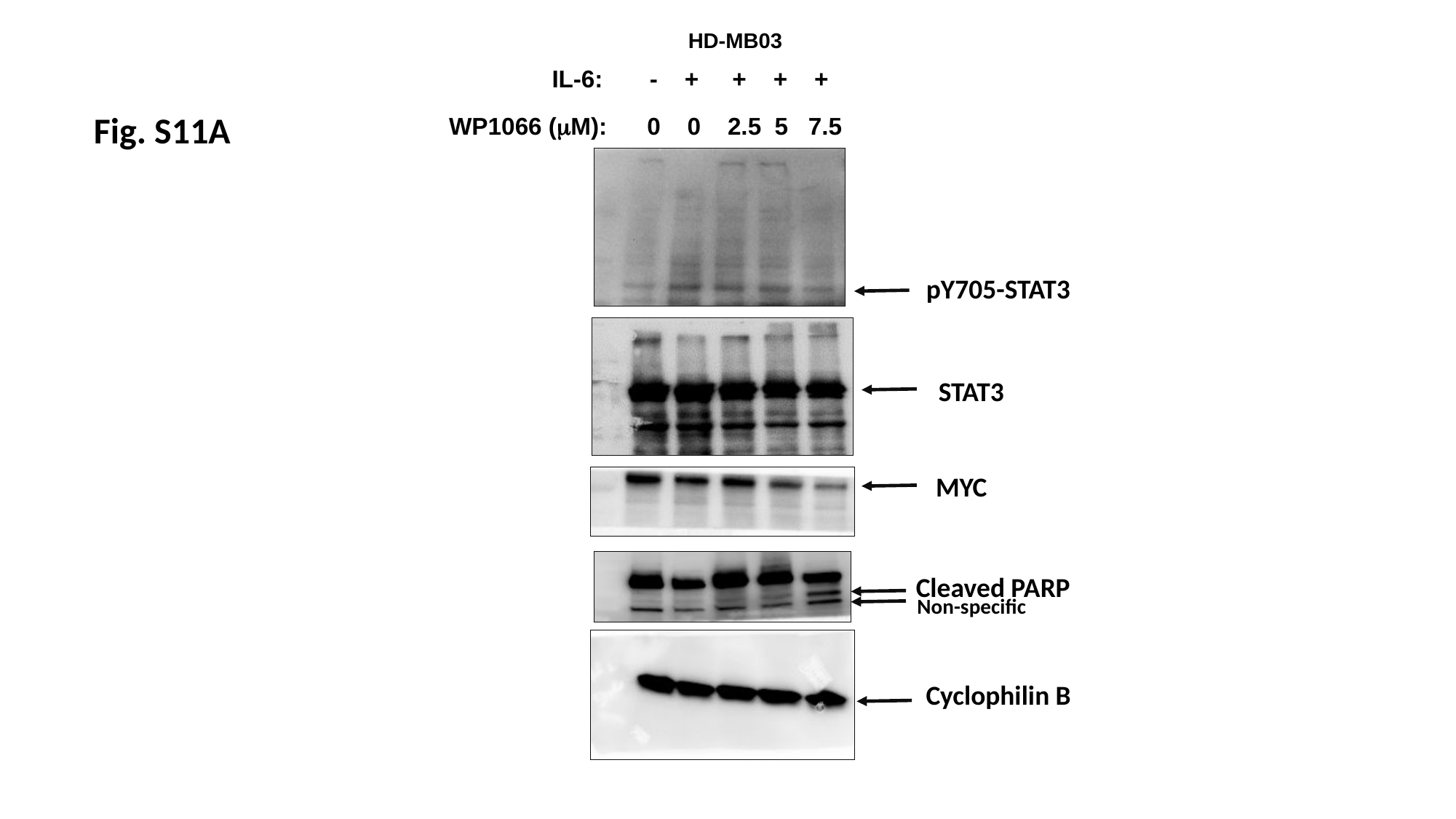

HD-MB03
IL-6: - + + + +
Fig. S11A
WP1066 (mM): 0 0 2.5 5 7.5
pY705-STAT3
STAT3
MYC
Cleaved PARP
Non-specific
Cyclophilin B
